# m2ST: dual multi-scale graph clustering for spatially resolved transcriptomics

**DOI:** 10.1093/bioinformatics/btaf221

**Published:** 2025-04-24

**Authors:** Wei Zhang, Ziqi Zhang, Hailong Yang, Te Zhang, Shu Jiang, Ning Qiao, Zhaohong Deng, Xiaoyong Pan, Hong-Bin Shen, Dong-Jun Yu, Shitong Wang

**Affiliations:** The School of Artificial Intelligence and Computer Science, Nantong University, Nantong, 226019, China; The School of Artificial Intelligence and Computer Science, Jiangnan University, Wuxi, 214122, China; The School of Artificial Intelligence and Computer Science, Jiangnan University, Wuxi, 214122, China; The School of Artificial Intelligence and Computer Science, Jiangnan University, Wuxi, 214122, China; The Lab for Uncertainty in Data and Decision Making (LUCID), School of Computer Science, University of Nottingham, Nottingham, NG81BB, United Kingdom; The School of Artificial Intelligence and Computer Science, Nantong University, Nantong, 226019, China; The School of Artificial Intelligence and Computer Science, Jiangnan University, Wuxi, 214122, China; The School of Artificial Intelligence and Computer Science, Jiangnan University, Wuxi, 214122, China; Department of Automation, Shanghai Jiao Tong University, Shanghai, 200240, China; Department of Automation, Shanghai Jiao Tong University, Shanghai, 200240, China; School of Computer Science and Engineering, Nanjing University of Science and Technology, Nanjing, 210094, China; The School of Artificial Intelligence and Computer Science, Jiangnan University, Wuxi, 214122, China

## Abstract

**Motivation:**

Spatial clustering is a key analytical technique for exploring spatial transcriptomics data. Recent graph neural network-based methods have shown promise in spatial clustering but face notable challenges. One significant issue is that analyzing the functions and complex mechanisms of organisms from a single scale is difficult and most methods focus exclusively on the single-scale representation of transcriptomic data, potentially limiting the discriminative power of extracted features for spatial domain clustering. Furthermore, classical clustering algorithms are often applied directly to latent representation, making it a worthwhile endeavor to explore a tailored clustering method to further improve the accuracy of spatial domain annotation.

**Results:**

To address these limitations, we propose m2ST, a novel dual multi-scale graph clustering method. m2ST first uses a multi-scale masked graph autoencoder to extract representations across different scales from spatial transcriptomic data. To effectively compress and distill meaningful knowledge embedded in the data, m2ST introduces a random masking mechanism for node features and uses a scaled cosine error as the loss function. Additionally, we introduce a tailored multi-scale clustering framework that integrates scale-common and scale-specific information exploration into the clustering process, achieving more robust annotation performance. Shannon entropy is finally utilized to dynamically adjust the importance of different scales. Extensive experiments on multiple spatial transcriptomic datasets demonstrate the superior performance of m2ST compared to existing methods.

**Availability and implementation:**

https://github.com/BBKing49/m2ST.

## 1 Introduction

In recent years, spatial transcriptomics technologies such as seqFISH+ (Eng *et al.* 2019), MERFISH (Zhang *et al.* 2020), and Slide-seqV2 (Stickels *et al.* 2021) have emerged as cutting-edge tools for understanding cellular dynamics and their in-situ microenvironments. Unlike single-cell RNA sequencing, spatial transcriptomics captures both gene expression and spatial location information, enabling deeper insights into molecular communication and tissue structure (Cheng *et al.* 2023).

Accurate spatial domain annotations form the foundation for subsequent functional studies. To this end, using clustering methods to annotate spatial transcriptomics data has become a popular research direction in recent years. In addition to the classical K-means (Hartigan and Wong 1979), Louvain (Blondel *et al.* 2008), and Leiden (Traag *et al.* 2019) are used to partition the spatial domain. Some researchers have developed some methods that use distance computation or probability estimation for clustering modeling. For example, based on Markov random fields, Dries *et al.* clustered spatial domains by comparing intrinsic gene expression in neighboring cells (Dries *et al.* 2021). Based on the Bayesian statistical method and the prior knowledge of spatial domains, Zhao *et al.* achieved the partition of spatial domains (Zhao *et al.* 2021). Yang *et al.* introduced Hidden Markov Random Fields to explore spatial dependencies and the associations between neighboring cells (Yang *et al.* 2022).

All of the methods mentioned above are based on traditional machine learning, while they ignore most of the valuable spatial coordinate information. To address it, several deep learning-based graph clustering methods for spatial transcriptomics data have been proposed. For instance, Pham *et al.* realized spatial domain clustering by integrating gene expression normalization, spatial location and morphological adjustments (Pham *et al.* 2020). Hu *et al.* introduced an undirected weighted graph to represent the dependencies of spatial data and extracted hidden embedding by using a graph convolutional network, and finally spatial domain partition by using the Iterative clustering method (Hu *et al.* 2021). Similarly, Xu *et al.* introduced a denoising auto-encoder and variational graph autoencoder to jointly learn hidden embedding for spatial domain clustering (Xu *et al.* 2022). Li *et al.* extracted hidden embedding of spatial transcriptomics data by using Deep Graph Infomax (Velickovic *et al.* 2019) and then introduced UMAP (Becht *et al.* 2018) to realize spatial domain partition (Li *et al.* 2022). Fang *et al.* introduced an adversarial graph autoencoder and pseudo-label learning mechanism for spatial domain clustering (Fang *et al.* 2024c). Moreover, some methods found that introducing a masking mechanism into the model can improve the discriminability of the learned hidden embedding. For example, Fang *et al.* introduced a masking mechanism and contrastive learning into graph autoencoder for spatial domain partition (Fang *et al.* 2024b). Min *et al.* proposed a dual-channel masked graph autoencoder for spatial domain partition (Min *et al.* 2024). Similarly, Fang *et al.* introduced triplet learning into masked graph autoencoder to further improve the discriminative of hidden embedding and achieved great clustering performance (Fang *et al.* 2024a).

While these deep learning-based graph clustering methods have demonstrated effectiveness, significant challenges remain. *First*, in the field of life sciences, understanding the complexity of organisms presents a significant challenge, as this complexity stems from their hierarchical structures and multidimensional interactions. Researchers are increasingly recognizing that relying solely on observations and analyses from a single level of inquiry often proves insufficient for gaining a comprehensive understanding of the organism’s functions and intricate mechanisms (Lagasse and Levin 2023, Ruscone *et al.* 2023). Meanwhile, some studies suggest that exploring multi-scale information enables a more comprehensive capture of data distributions, thereby enhancing the robustness of models (Li *et al.* 2015, Somnath *et al.* 2021). However, existing spatial domain annotation methods are limited to extracting latent representations with a single scale, which often fails to ensure that the representations contain sufficient discriminative information for accurate spatial domain partition. Therefore, how to design a novel graph neural network to explore the multi-scale information within spatial transcriptomics data is a meaningful research issue. *Second*, current methods apply classical clustering methods directly to the learned representations for spatial domain annotation, which cannot fully leverage the rich information embedded in the latent representations. Consequently, designing a clustering method tailored to the spatial transcriptomic representation extraction is significant to further enhance clustering performance and achieve more accurate spatial domain annotation.

To address above limitations, we propose m2ST, a novel dual multi-scale graph clustering method for spatial transcriptomics data. The proposed m2ST consists of a multi-scale graph masked autoencoder (MC_GMAE) and a multi-scale clustering method. Specifically, we first propose a novel self-supervised multi-scale graph masked autoencoder (MC_GMAE) based on the Graph Attention Network (GAT) (Veličković *et al.* 2017) to explore the spatial transcriptomics data from different scales. In the encoder network of MC_GMAE, which consists of a shared GAT layer and multiple specific GAT layers. The shared layer is used for primary information exploration and multiple specific layers are used to explore knowledge at different scales. Corresponding multiple specific GAT layers for different scales make up the decoder network, which enhances the representation of spatial information across varying levels of granularity. Meanwhile, to further extract and condense knowledge within the spatial transcriptomics data, we further introduce a feature-masked mechanism that randomly replaces a subset of node features in the encoder and decoder with learnable vectors. In addition, a scaled cosine error loss function is introduced to further enhance the robustness of the model. To enable more targeted clustering of the extracted multi-scale representations and further enhance the accuracy of spatial domain annotation, we propose a novel multi-scale clustering method. In this method, a dual representation learning mechanism based on matrix factorization is first constructed to explore scale-common and scale-specific knowledge across scales. Meanwhile, we integrate dual representation learning with clustering partitioning into a unified framework, allowing these two parts to mutually reinforce and enhance each other. Finally, we introduce Shannon entropy to adaptively adjust the importance of different representations.

We comprehensively evaluated the proposed method m2ST on five spatial transcriptomics datasets. Experimental results demonstrate that m2ST significantly outperforms state-of-the-art methods, proving its robustness in identifying spatial domains and its potential applicability to broader spatial transcriptomics datasets.

## 2 Materials and methods

### 2.1 The overview of the proposed m2ST

**Figure 1.**
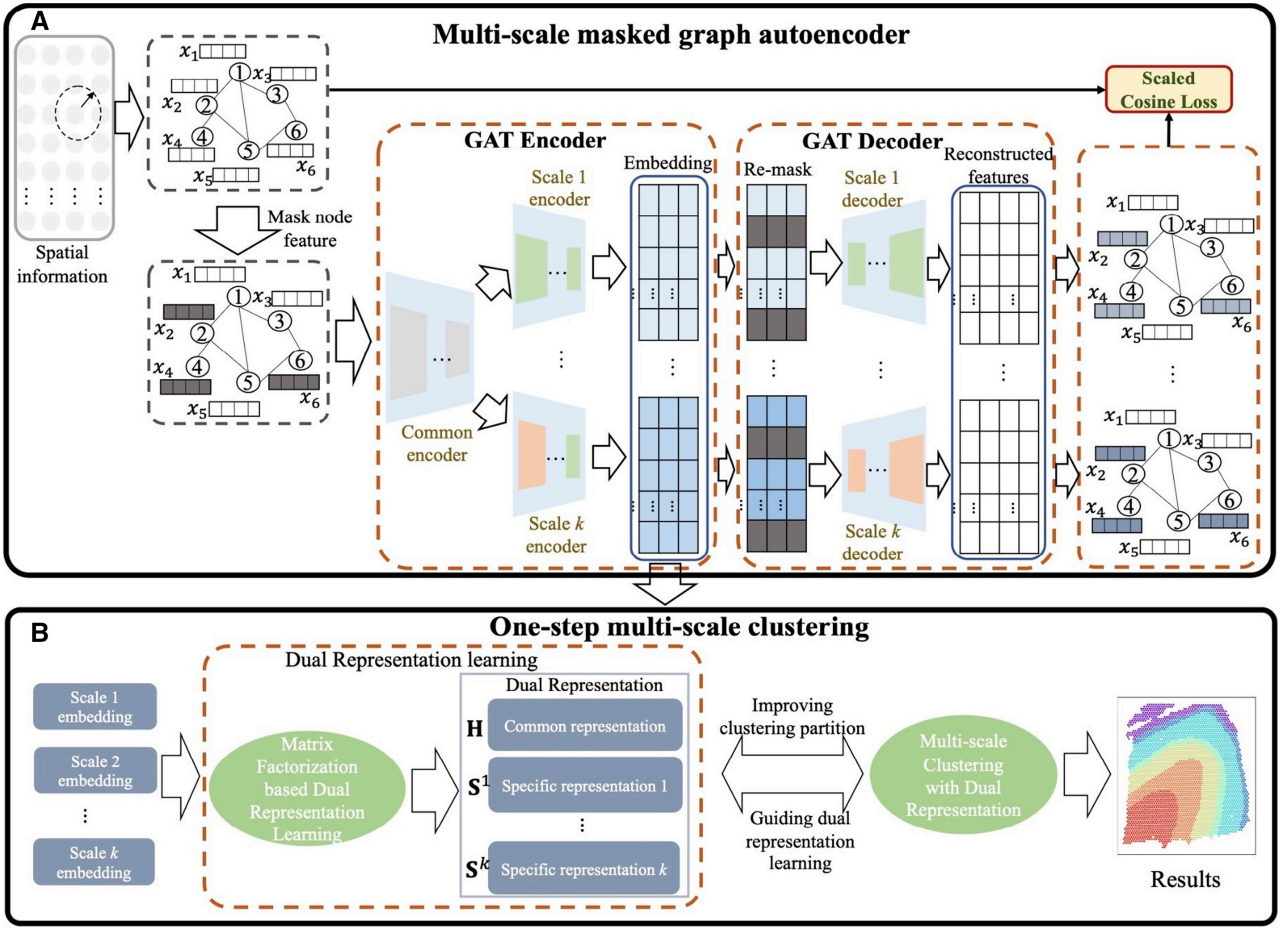
The framework of the proposed m2ST. Part A is the multi-scale masked graph auroencoder and Part B is the one-step multi-scale clustering.

To comprehensively analyze spatial transcriptomics data, we propose a multi-scale spatial transcriptomics clustering framework, as illustrated in Fig. 1. This framework consists of two main components:

#### 2.1.1 *Part A: multi-scale masked graph autoencoder*

First, we construct a graph G=(V,A,X), to to express spatial transcriptomics data, where V is the set of nodes, $A\in R^{N\times N}$ is the adjacency matrix, and $X\in R^{d\times N}$ is the node identity matrix. *N* is the number of nodes and *d* is the feature dimension. Details of the graph construction process are provided in the Supplementary Part S1.

To fully extract graph embeddings, we design a novel multi-scale masked graph autoencoder based on Graph Attention Networks (GAT). This autoencoder incorporates a masking mechanism that randomly replaces parts of the input feature matrix with learnable vectors. This strategy mitigates common trivial solutions encountered in autoencoders and enhances robustness. Specifically, the masked graph data is input into a multi-scale encoder, which is composed of a shared encoder and several scale-specific encoders to extract multi-scale latent embedding. Further, the obtained embeddings are re-masked and passed into a multi-scale decoder to enhance both encoder and decoder learning ability. In the decoder, we tailored distinct decoders for each scale of the hidden embeddings to achieve feature reconstruction. To guide the learning process, we introduce a scaled cosine loss function that evaluates the reconstruction error between the original and reconstructed features. This enables effective self-supervised learning and ensures that the embeddings are both discriminative and informative.

#### 2.1.2 *Part B: multi-scale clustering*

Based on the learned multi-scale embeddings, we develop a novel multi-scale clustering method to partition spatial domains. First, a dual representation learning method is introduced to explore scale-common and scale-specific knowledge from the multi-scale embeddings. Then, a unified clustering framework is constructed to integrate dual representation learning and clustering process. Finally, the Shannon entropy is introduced to balance the importance of different scales. By tightly coupling dual representation learning and clustering, the framework achieves optimal clustering performance.

Detailed descriptions of Part A and B are provided in the subsequent parts.

### 2.2 Multi-scale masked graph autoencoder

#### 2.2.1 *Node feature-masked mechanism*

Autoencoder is the classic model consisting of an encoder and decoder, which extracts discriminative embedding through two primary steps: information compression and reconstruction. However, traditional autoencoders may converge to trivial solutions (Hou *et al.* 2022), and existing graph autoencoders that target feature reconstruction usually ignore this issue. To address it, denoising autoencoder (Vincent *et al.* 2008) alleviates the trivial solution problem by corrupting the input data. Meanwhile, the feature-masked mechanism has been applied in some neural networks and spatial transcriptomics data analysis (Hou *et al.* 2022, 2023, Fang *et al.* 2024b, Min *et al.* 2024). Following these successful experiences. In this paper, we also introduce a feature-masked mechanism and construct a multi-scale masked graph autoencoder. We randomly select $\tilde{V}\subset V$ from the set of nodes V and mask each of their features with a learnable vector $X_{i,[M]}\in R^{d\times 1}$. Thus, the masked feature matrix $\tilde{X}$ can be defined as:


(1) X∼i={X[Masked], Vi∈V∼Xi,       Vi∉V∼

Thus, the goal of the proposed multi-scale masked graph autoencoder is to reconstruct the masked features of nodes in $\tilde{V}$ based on partially observed node features $\tilde{X}$ and the input adjacency matrix A. Therefore, the graph data input to the multiscale shielded graph autoencoder is $G=(V,A,\tilde{X})$. It is important to note that the masking operation is applied only during training. During testing, the encoder directly extracts the embeddings without masking.

#### 2.2.2 *Multi-scale encoder*

The Graph Attention Network achieves powerful graph data exploitation ability and has received widespread attention in recent years by introducing the multi-head attention mechanism into graph neural networks. Thus, to extract high-discriminative spatial transcriptomic features with different scales, we use GAT as the foundational model. As shown in Fig. 1A, the constructed multi-scale encoder consists of two parts: the first part is a shared encoder ${\tilde{X}}_{se}=f_{E}(A,\tilde{X})$, which provides an initial representation learning of spatial transcriptomic data. The second part is the scale-specific encoder $H^{m}=f_{E\_m}(A,{\tilde{X}}_{se})$ to explore the information from different scales. In the shared encoder ${\tilde{X}}_{se}=f_{E}(A,\tilde{X})$, which is consistent with the GAT. We first compute the attention coefficients for each node in the graph. Then, based on these coefficients, a weighted sum is performed to obtain the updated features for each node. The flowchart of GAT is provided in the Supplementary Fig. S1. A detailed explanation is given below, starting with the computation method for the attention coefficients:


(2) $$a_{i,j}=\frac{\;\text{exp}\;(\mathrm{LeakyReLU}(e_{i,j}))}{\sum_{l}^{V_{i}}\;\text{exp}\;(\mathrm{Leak}y\mathrm{ReLU}(e_{i,l}))}$$

(3) $$e_{i,j}=g([W{\tilde{X}}_{i}||W{\tilde{X}}_{j}];\theta )$$

(4) LeakyReLU(ei,j)={ei,j if ei,j≥0ϵei,j if ei,j<0

where $a_{i,j}$ is the attention coefficient obtained by normalizing the similarity scores of all adjacent nodes. $V_{i}$ is the set of neighboring nodes obtained from the adjacency matrix A. LeakyReLU(*) is the activation function and ϵ=0.01. $e_{i,j}$ is the similarity coefficient of node *i* and *j*, $W\in R^{d^{\prime }\times d}$ is the shared mapping matrix, $d^{\prime }$ is the dimension of mapped space. ${\tilde{X}}_{i}\in R^{d\times 1}$ is the feature vector of the *i*th node, || is the splice operation. Following (Hou *et al.* 2022), g(*) is set as a single layer feedforward network and θ is the learnable parameters. Similar to (3), we can compute *K* coefficients $a_{i,j}^{k},\;k=1,2,\ldots ,K$, as multi-head attention coefficients. When the multi-head attention coefficients have obtained, we update the features of each node using the multi-head attention mechanism on the graph:


(5) $${\tilde{X}}_{i,se}=\sigma (\sum_{k}^{K}\sum_{j}^{V_{i}}a_{i,j}^{k}W^{k}{\tilde{X}}_{i})$$

where $a_{i,j}^{k}$ and $W^{k}$ are the attention coefficients and shared mapping matrix of the *k*th head, respectively. σ(*) is the PreLU activate function to enhance the flexibility of the network (He *et al.* 2015). ${\tilde{X}}_{i,se}\in R^{d^{\prime }\times 1}$ is the updated feature vector of the *i*th node with shared encoder.

Existing studies have shown that exploring multi-scale information can provide a more comprehensive capture of data distribution knowledge (Li *et al.* 2015, Somnath *et al.* 2021). Therefore, based on GAT, we constructed multi-scale specific encoders to explore the information with different granularities. Denoting the extracted *m*th embedding $H^{m}=f_{E\_m}(A,{\tilde{X}}_{se})$, where $f_{E\_m}(*)$ denotes the *m*th encoder. Similar to $f_{E}(*)$, the details are as follows:


(6) $$a_{i,j}^{m,k}=\frac{\;\text{exp}\;(\mathrm{LeakyReLU}(f(\left[W_{m}^{k}{\tilde{X}}_{i,se}||W_{m}^{k}{\tilde{X}}_{j,se}\right])))}{\sum_{l}^{V_{i}}\;\text{exp}\;(\mathrm{LeakyReLU}(f(\left[W_{m}^{k}{\tilde{X}}_{i,se}||W_{m}^{k}{\tilde{X}}_{l,se}\right])))}$$

(7) $$H_{i}^{m}=\sigma (\sum_{k}^{K}\sum_{j}^{V_{i}}a_{i,j}^{m,k}W^{m,k}{\tilde{X}}_{i,se})$$

where $W^{m,k}\in R^{d_{m}^{\prime }\times d^{\prime }}$ and $a_{m,i,j}^{k}$ are the mapped matrix and attention coefficient of the *k*th head at the *m*th scale, respectively. $H_{i}^{m}\in R^{d_{m}^{\prime }\times 1}$ is the mapped feature vector of the *i*th node with the *m*th encoder.

#### 2.2.3 *Multi-scale decoder*

After obtaining the multi-scale embeddings compressed by the encoder, we construct a re-masked decode by following (Fang *et al.* 2024b, Hou *et al.* 2023, Min *et al.* 2024) generalize to multi-scale decoder learning and further enhance the robustness of the decoder. We apply another set of masks to replace the previously masked node indices in $H^{m}$, i.e. ${\tilde{H}}^{m}=\mathrm{Remask}(H^{m})$. Similar to (1), ${\tilde{H}}^{m}$ is defined as follows:


(8) H∼im={H[Masked]m, Vi∈V∼Him,      Vi∉V∼

This method compels the decoder to reconstruct the masked representations from adjacent unmasked representations, which can further enhance the robustness of the autoencoder (Hou *et al.* 2023). We also use a single-layer GAT as the decoder for each scale data. This method allows the model to recover the feature of a node based on a group of nodes, rather than relying solely on the node itself, thereby supporting the encoder in learning high-discriminative embeddings, i.e. $Z^{m}=f_{D\_m}(A,{\tilde{H}}^{m})$, where $f_{D\_m}(*)$ denotes the *m*th encoder. The details are as follows:


(9) $$Z_{i}^{m}=\sigma (\sum_{k}^{K}\sum_{j}^{V_{i}}b_{i,j}^{m,k}Q^{m,k}{\tilde{H}}_{i}^{m}),\;i=1,2,\ldots ,N$$

where $Q^{m,k}\in R^{d\times d_{m}^{\prime }}$ and $b_{i,j}^{m,k}$ is the mapped matrix and attention coefficient of the *k*th head at the *m*th scale decoder, respectively. $Z_{i}^{m}\in R^{d\times 1}$ is the feature vector reconstructed by the decoder at different scales.

#### 2.2.4 *Loss function*

In traditional autoencoders, mean squared error (MSE) is typically used as the loss function (Wang *et al.* 2017, Park *et al.* 2019, Jin *et al.* 2020). To achieve a more robust model, we introduce scaled cosine error as the loss function by following (Hou *et al.* 2022), which is shown below:


(10) $$L_{\mathrm{SCE}}=\frac{1}{M}\sum_{m}^{M}\frac{1}{\left|\tilde{V}\right|}\sum_{i}^{\tilde{V}}{\left(1-\frac{X_{i}^{T}Z_{i}^{m}}{{\left\|X_{i}\right\|}_{2}\cdot {\left\|Z_{i}^{m}\right\|}_{2}}\right)}^{\gamma }$$

where $X_{i}$ and $Z_{i}^{m}=f_{D\_m}(A,f_{E\_m}(A,f_{E}(A,{\tilde{X}}_{i})))$ are the original and reconstructed features. γ≥1 is the scaling factor and serves as a hyperparameter. The $L_{2}$-norm in the scaled cosine error maps vectors onto a unit hypersphere, effectively enhancing the stability of representation learning during training (Grill *et al.* 2020).

### 2.3 Multi-scale clustering

#### 2.3.1 *Dual representation learning mechanism for multi-scale spatial transcriptomics embedding*

Based on the above multi-scale masked graph autoencoder, we obtained multi-scale hidden embeddings $H^{m}$ of spatial transcriptomics data, and traditional strategies can be used to combine embeddings from different scales for clustering. However, since each scale represents the data at a different granularity while sharing the same objective, the multi-scale embeddings—similar to multi-view data—contain both shared knowledge across scales and unique information specific to each scale (Zhang *et al.* 2024). Therefore, a more effective mechanism is required to explore both types of knowledge efficiently. Denoting $H^{m}={\left(H^{m}\right)}^{T},\;m=1,\;2,\ldots ,\;M$ and based on the matrix factorization technique, we propose a dual representation learning mechanism to explore the common and scale-specific information from multi-scale data simultaneously as follows:


(11) $$\begin{array}{c} \underset{H_{c},W^{m},H_{s}^{m},P^{m}}{\min}\sum_{m}^{M}{\left\|H^{m}-H_{c}^{T}W^{m}-H_{s}^{m,T}P^{m}\right\|}_{F}^{2}+\beta {\left\|H_{c}^{T}\right\|}_{F}^{2}+\;\\ \beta \sum_{m}^{M}{\left\|H_{s}^{m,T}\right\|}_{F}^{2} \end{array}$$

where $H_{c}\in R^{d_{c}\times N}$ is the common latent representation. $H_{s}^{m}\in R^{d_{s}\times N}$ is the specific representation of the *m*th scale. $d_{c}$ and $d_{s}$ are the feature dimension of the common and specific representation. $W^{m}\in R^{d_{c}\times d_{m}^{\prime }}$ and $P^{m}\in R^{d_{s}\times d_{m}^{\prime }}$ is the mapping matrix of *m*th scale and β is regularization parameters. Furthermore, to enable the learned representations to be more robust, regularization terms are introduced.

#### 2.3.2 *Objective function for multi-scale clustering*

In the previous section, we extract the common and specific knowledge among different scales, and to make the learned knowledge more suitable for clustering partition, a new unified clustering framework is proposed by introducing Shannon entropy mechanism and orthogonal constraint. Denoting the common representation as the *M *+* *1 scale. The proposed objective function is given as follows:


(12) $$\begin{array}{c} \underset{H_{c},W^{m},H_{s}^{m},\;P^{m},U,V^{m},\alpha^{m}}{\min}\sum_{m}^{M}{\left\|H^{m}-H_{c}^{T}W^{m}-H_{s}^{m,T}P^{m}\right\|}_{F}^{2}+\beta {\left\|H_{c}^{T}\right\|}_{F}^{2}+\;\\ \begin{array}{c} \beta \sum_{m}^{M}{\left\|H_{s}^{m,T}\right\|}_{F}^{2}+\sum_{m}^{M}\alpha^{m}{\left\|H_{s}^{m}-V^{m}U\right\|}_{F}^{2}+\alpha^{M+1}{\left\|H_{c}-V^{M+1}U\right\|}_{F}^{2}+\;\\ \begin{array}{c} \lambda \sum_{m}^{M+1}{\left\|V^{m,T}V^{m}-I\right\|}_{F}^{2}+\delta \sum_{m}^{M+1}\alpha^{m}ln\alpha^{m}\\ s.t.\;\alpha^{m}\geq 0,\sum_{m}^{M+1}\alpha^{m}=1,U_{i,j}\in \{0,1\},\sum_{i=1}^{C}U_{i,j}=1 \end{array} \end{array} \end{array}$$

where the fourth and fifth terms are clustering terms (Hartigan and Wong 1979), $V^{M+1}\in R^{d_{c}\times C}$ and $V^{m}\in R^{d_{s}\times C}$ (m=1,2,…,M) are the clustering center of the common and specific representations, respectively. *C* is the number of clusters. $U\in R^{C\times N}$ is the cluster indicator matrix. When the *j*th instance is clustered into the *i*th class, $U_{i,j}=1$, otherwise, $U_{i,j}=0$. $\alpha^{m}$ is the weight for different representations. $I\in R^{C\times C}$ is the identity matrix. β,λ,δ≥0 are parameters. In (12), the interplay between the extracted common and scale-specific representations and the learned cluster indicator matrix fosters a reciprocal enhancement process. The representations improve clustering performance, while the cluster indicator matrix enhances the discriminatory power of the representations. The detailed description and optimization process for (12) is presented in the Supplementary Part S2.

Based on the above description and analysis, the algorithm description of m2ST is given in the Supplementary Part S3.

## 3 Results

### 3.1 Experiments setting

#### 3.1.1 *Datasets*

We conduct extensive experiments on six datasets [DLPFC (Pardo *et al.* 2022), Breast cancer (Xu *et al.* 2024), STARmap (Wang *et al.* 2018), Mouse hippocampus (Palla *et al.* 2022), Mouse cerebellum (Rodriques *et al.* 2019, Shang and Zhou 2022), and Human Heart (Xue *et al.* 2024)]. These data cover a range of sample sizes from several thousand to several tens of thousands, facilitating a comprehensive evaluation of the performance and scalability of our method. The statistics and detailed described of these data are given in Supplementary Part S4.

#### 3.1.2 *Evaluation metrics and parameters setting*

To comprehensively verify the effectiveness of m2ST, we follow (Cai *et al.* 2013) and adopt NMI, ARI, and Purity as evaluation metrics, where higher values indicate better performance. Meanwhile, for datasets without ground truth, the Silhouette Coefficient (SC) (Scrucca *et al.* 2016) and Davies–Bouldin (DB) (Blondel *et al.* 2008) are used as evaluation metrics. Specifically, a higher SC value represents better clustering performance, while a lower DB value indicates better performance. In addition, detailed parameters setting and evaluation metrics are given in Supplementary Part S4.

### 3.2 Experimental results

#### 3.2.1 *Experimental results and analyses on the DLPFC dataset*

**Figure 2.**
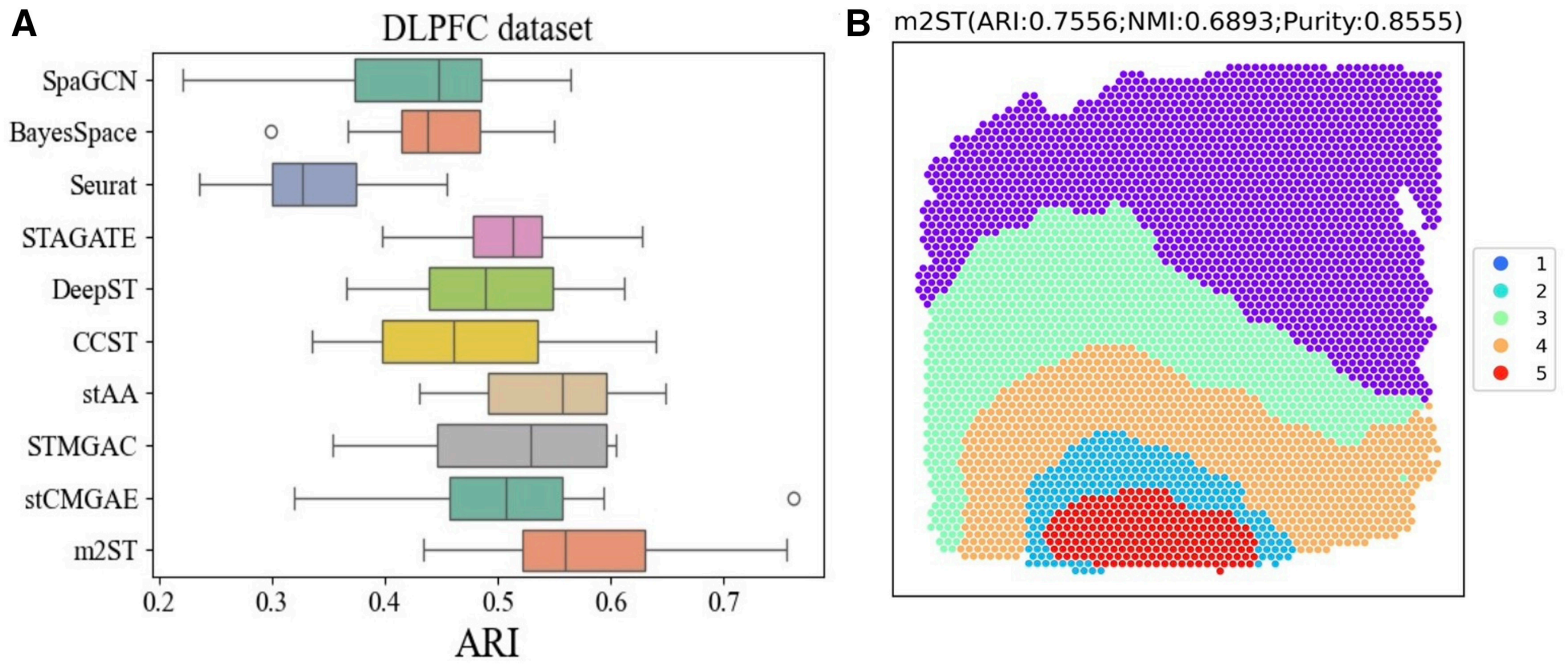
Comparison results on the DLPFC. (A) Clustering results for all methods on ARI metric. (B) Visualization results of m2ST on slice 151672.

First, we compared m2ST with nine state-of-the-art spatial clustering methods [SpaGCN (Hu *et al.* 2021), DeepST (Xu *et al.* 2022), BayesSpace (Zhao *et al.* 2021), Seruat (Hao *et al.* 2021), STAGATE (Dong and Zhang 2022), CCST (Li *et al.* 2022), stAA (Fang *et al.* 2024c), STMGAC, and stCMGAE] on the DLPFC dataset. This dataset consists of 12 slices, and we conducted a comprehensive comparison across all 12 slices. Figure 2A shows the results of all methods on the ARI metric, and these results of all methods are given in Supplementary Fig. S2. From the results, it can be seen that m2ST consistently outperforms others across all three metrics, with a particularly significant advantage in the ARI metric. Compared to graph autoencoder based methods (SpaGCN, stAA, CCST, DeepST), the performance of m2ST is the best, suggesting that exploring the multi-scale information simultaneously is effective. Meanwhile, Fig. 2B presents the visualization results of m2ST on 151672 slices, and rest visualization results of all the methods are also given in Supplementary Fig. S3. From Fig. 2B and Supplementary Fig. S3, it can be seen that the clustering delineation of the proposed m2ST method is the best, indicating its ability to accurately identify the spatial domain structure of cells. Notably, compared to other methods, m2ST demonstrates optimal recognition performance for the case of Layer_3. Finally, we present the UMAP plot and PAGA trajectory inference (Wolf *et al.* 2019) results of the proposed m2ST in Supplementary Fig. S3B and C. The UMAP result clearly shows that each layer exhibits distinct regions, indicating that the m2ST method effectively separates domains across different layers. Moreover, the PAGA graph reveals a linear trajectory from WM to Layer 3, demonstrating that the developmental trajectory inferred by m2ST aligns with the spatial topology of the slice.

#### 3.2.2 *Comparison of the proposed method on breast cancer, STARmap datasets with SOTA spatial clustering methods*

**Figure 3.**
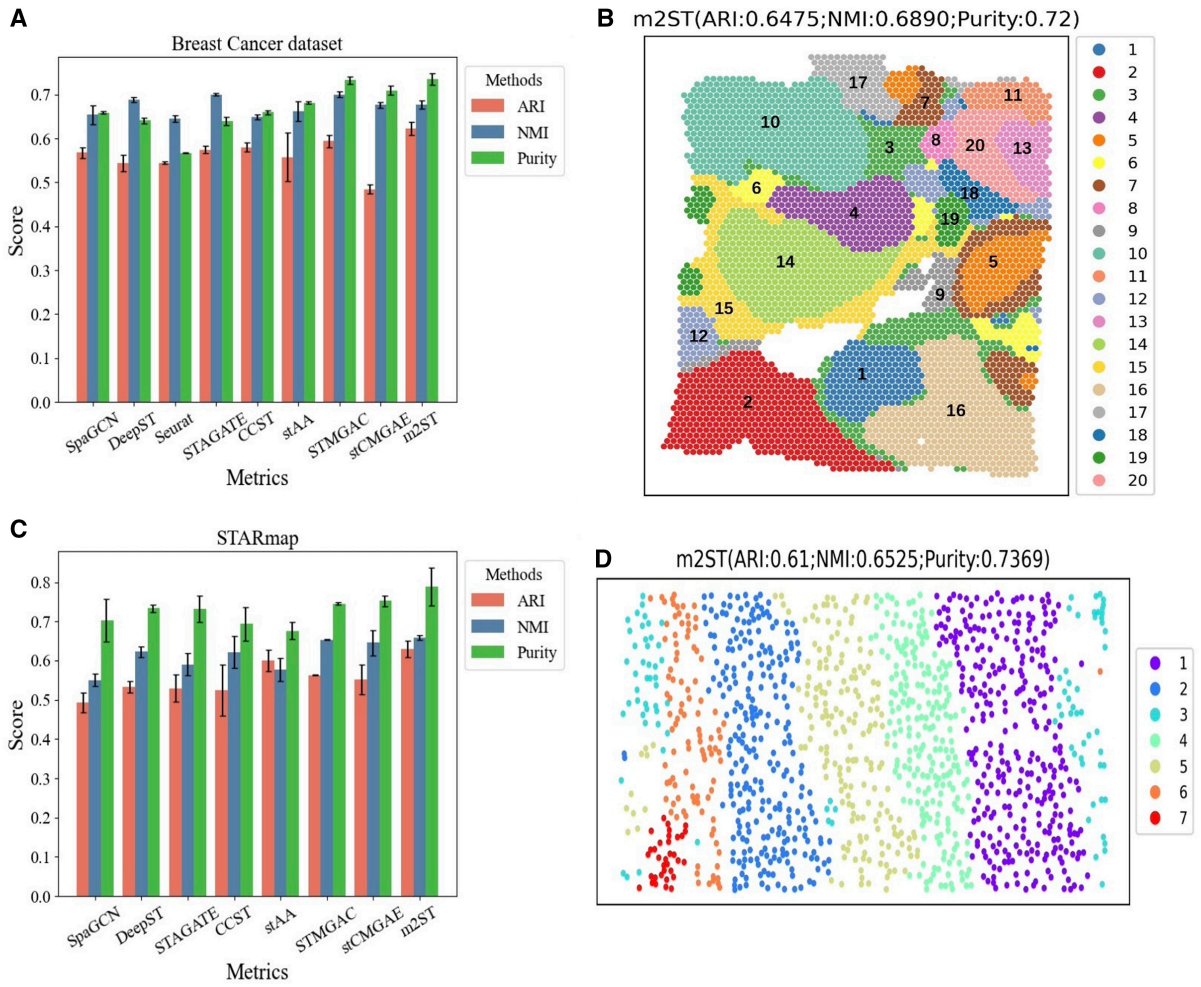
Comparison results on the Breast cancer and STARmap dataset. (A) and (B) Clustering results and visualization results on the Breast cancer. (C) and (D) Clustering results and visualization results on STARmap dataset.

Subsequently, we conducted comparisons and analyses with several advanced methods on two representative labeled datasets (Breast cancer and STARmap datasets). First, Fig. 3A shows the spatial domain partition results on the Breast Cancer dataset. Second, Fig. 3B and Supplementary Fig. S4 present the visualization results of the all methods. The visualizations show that m2ST achieves better spatial domain segmentation results, with many clusters aligning well with manual annotations, such as the Tumor_edge_2, DCIS/LCIS, IDC_2, IDC_4, and IDC_5 domains. Specifically, for the IDC_5 domain, methods such as stCMGAE, CCST and stAA clustered it into multiple domains.

We further validated the effectiveness of the proposed m2ST method on the STARmap dataset. First, Fig. 3C shows the clustering results of eight methods on three metrics. Meanwhile, Fig. 3D and Supplementary Fig. S5 presents the visualization results of all methods. From the results, it is evident that m2ST accurately partitions the STARmap dataset into seven spatial domains, providing a solid foundation for subsequent studies. Meanwhile, the results demonstrate that the proposed m2ST outperforms all other methods in terms of ARI. Although it is slightly weaker than CCST in NMI and stCMGAE in Purity, m2ST significantly surpasses CCST and stCMGAE in the other two metrics, which further confirms the benefits of exploring multi-scale information.

#### 3.2.3 *Comparison of the proposed method with the SOTA spatial clustering methods on mouse hippocampal, mouse cerebellum, and human heart datasets without ground truth*

**Figure 4.**
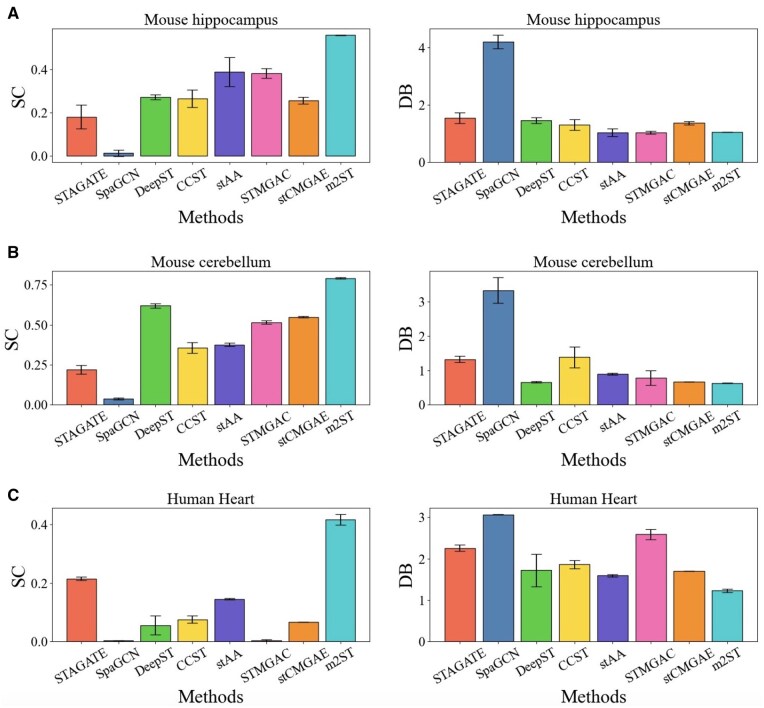
Clustering results on datasets without ground truth. (A) shows the results on the Mouse hippocampus dataset; (B) shows the results on the Mouse cerebellum dataset; and (C) shows the results on the Human heart dataset.

For unlabeled dataset, m2ST is compared with seven methods (STAGATE, SpaGCN, DeepST, CCST, stAA, STMGAC, and stCMGAE) on the unlabeled mouse hippocampus and mouse cerebellum dataset. First, we present the comparison with all methods on the mouse hippocampus dataset. The values of SC and DB metrics of these methods are given in Fig. 4A. It can be seen that the proposed m2ST outperforms the other methods on the SC metric, particularly surpassing SpaGCN by nearly 5%. At the same time, on the DB metric, the value of m2ST is much lower than the other methods (lower values indicate better performance), which further validates the effectiveness of our method on unlabeled datasets. Supplementary Figs S6 and S7 also shows the visualization results of all methods. From the Supplementary Fig. S7, it can be seen that m2ST has the clearest spatial delineation effect. Furthermore, the mouse hippocampus consists of three main regions: Cornu Ammonis 1 (CA1)/CA2, Cornu Ammonis 3 (CA3), and the Dentate Gyrus (DG), as shown in the first image of Supplementary Fig. S7 (Sunkin *et al.* 2013). From the Supplementary Fig. S7, it can be seen that m2ST demonstrates the ability to accurately identify these three regions.

Furthermore, the clustering results of eight methods on the mouse cerebellum and Human heart datasets are presented in Fig. 4B and C. Meanwhile, the visualization results of all methods are presented in Supplementary Figs S7 and S8. From the visualization results, m2ST, along with STMGAC, stCMGAE, and stAA, can accurately delineate the major regions of the mouse cerebellum and human heart. However, in terms of quantitative metrics, m2ST achieves a significantly higher SC value and a much lower DB value compared to other methods. Overall, these results demonstrate that m2ST exhibits superior clustering performance.

#### 3.2.4 *Unveiling cancer heterogeneity with the annotated breast cancer dataset*

Based on the clustering annotations of breast cancer, we explored clusters 14 and 15 to explore the cancer heterogeneity. We first performed differential gene expression analysis between these two clusters. As shown in Supplementary Fig. S10, genes such as COX6C, XBP1, and HSP90AB1 were highly expressed in cluster 14. Notably, COX6C and HSP90AB1 have been reported to promote cancer cell proliferation (Haase and Fitze 2016, Liu *et al.* 2024). Consequently, cluster 14 is identified as a malignant region, consistent with the ground truth labels, further demonstrating that m2ST can accurately localize cancerous areas.

**Figure 5.**
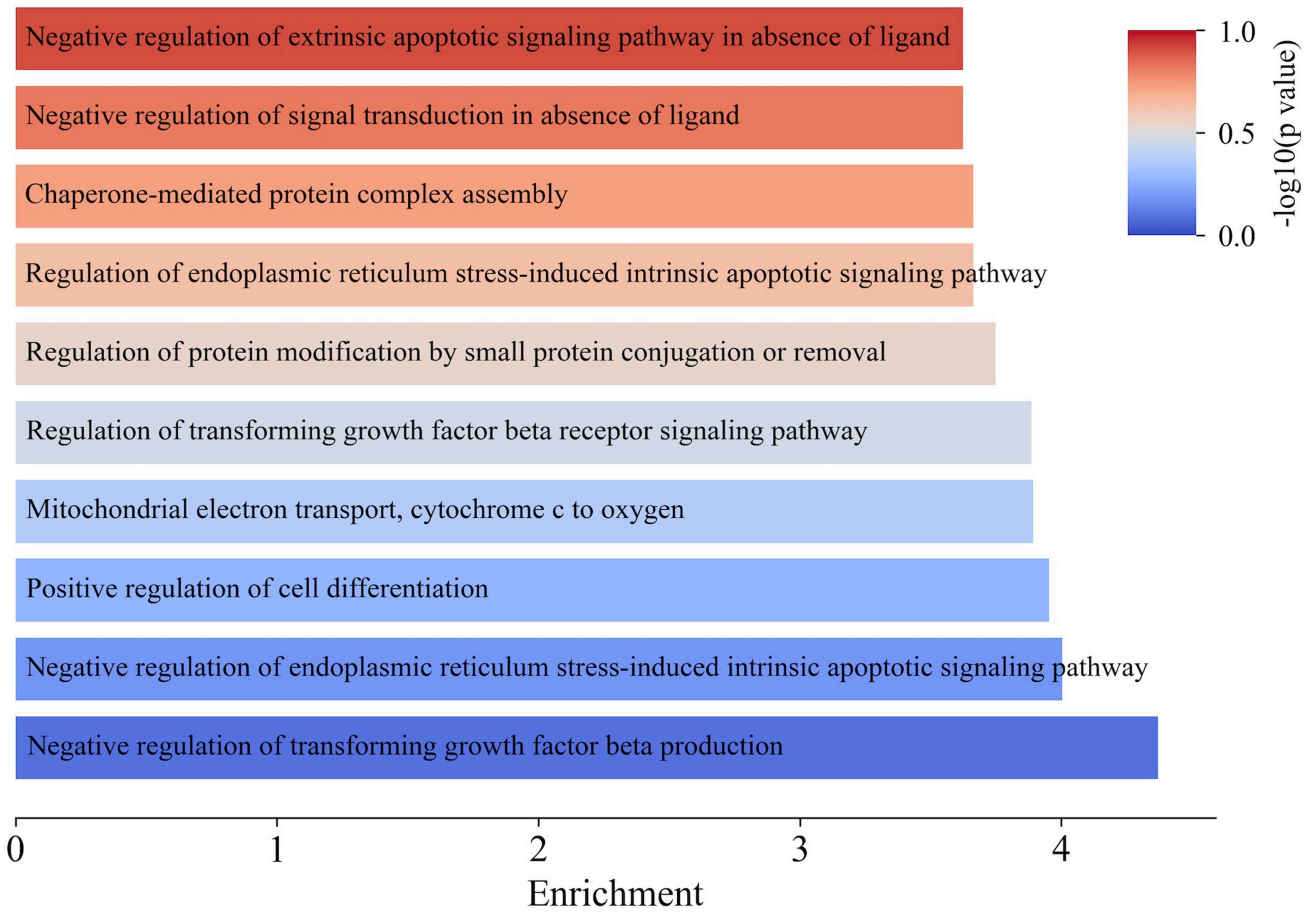
The enriched GO terms in cluster 14 of the Breast cancer dataset.

To gain deeper insights, we conducted a Gene Ontology (GO) enrichment analysis. Figure 5 presents the top five enriched GO terms in cluster 14, including negative regulation of transforming growth factor beta production, negative regulation of endoplasmic reticulum stress-induced intrinsic apoptotic signaling pathway, and mitochondrial electron transport, cytochrome c to oxygen. These results suggest that cluster 14 cells rapidly proliferate and exhibit enhanced immune evasion (Colak and Ten Dijke 2017, Schonthal 2012). Similarly, we analyzed cluster 15 and the analysis results are given in Supplementary Fig. S11. The results show an enrichment of GO terms such as T cell apoptotic process and regulation of Acyl-CoA biosynthetic process. This indicates that cluster 15 represents an immunosuppressive, tumor-promoting microenvironment, which may facilitate tumor progression and metastasis (Uzzo *et al.* 1999, Mashima *et al.* 2009). Therefore, m2ST effectively describes intratumoral heterogeneity within tumor regions, providing insights into both the growth state of cancer cells and the tumor-associated microenvironment at the tumor margin.

#### 3.2.5 *Unveiling neural spatial features with the annotated mouse cerebellum dataset*

**Figure 6.**
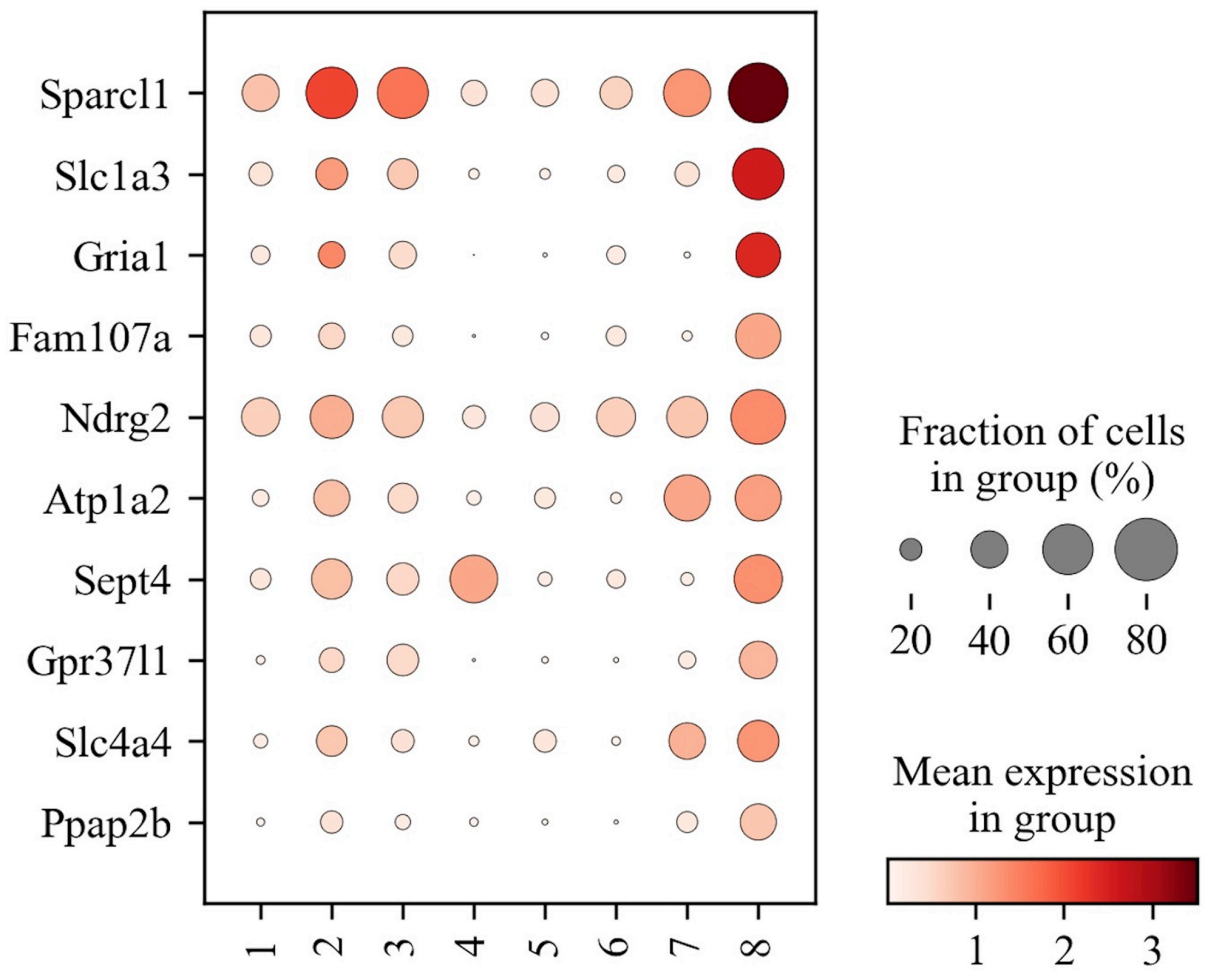
The differential expression genes of cluster 8 and other clusters of the Mouse cerebellum dataset.

We further analyzed the mouse cerebellum dataset to explore neural spatial features. We examined the differential gene expression between cluster 8 and other clusters. As shown in Fig. 6 the genes, i.e. Sparcl1, Slc1a3, and Gria1, are highly expressed in cluster 8. These genes are closely associated with neuronal functions and the regulation of glial cells in the nervous system (Kaczmarczyk *et al.* 2021, Bartelt *et al.* 2023). Meanwhile, according to (Shang and Zhou 2022), cluster 8 corresponds to the cerebellar granule layer, indicating that these genes are primarily enriched in cluster 8 rather than other clusters. Additionally, we conducted GO enrichment analysis (Wu *et al.* 2021) in Supplementary Fig. S6, and our analysis revealed that cluster 8 is significantly enriched in biological processes and cellular components related to ion transport and membrane functions, including GO terms such as *monoatomic ion transport*, *plasma membrane*, and *extracellular space*. Notably, these GO terms exhibit associations with the genes Gprin3 and Cemip. These results are consistent with those found in previous work (Rodriques *et al.* 2019). Specifically, these genes are novel spatially patterned genes, which are discovered by (Rodriques *et al.* 2019) and show specific localization to the granule layer. This further validates that m2ST has a strong capability for spatial transcription data mining.

### 3.3 Ablation studies

To further validate the effectiveness of the introduced multi-scale learning mechanism, masked mechanism, and multi-scale clustering method in the proposed m2ST, we conducted ablation experiments on three labeled datasets, i.e. DLPFC, Breast cancer, and STARmap. Denoting m2ST1 as the model with the masked mechanism, multi-scale learning, and multi-scale clustering removed, m2ST2 as the model with the masked mechanism and multi-scale clustering removed, m2ST3 as the model with multi-scale clustering removed, and m2ST4 as the model using the MSE loss. The experimental results on the ARI metric are presented in Table 1 and these results are given in Supplementary Table S2. Since the DLPFC dataset consists of 12 slices, we record results across all 12 slices and report the mean results for comparison. As shown in Table 1 and Supplementary Table S2, m2ST1 and m2ST4 is the worst in most cases, indicating that multi-scale learning and scaled cosine error can greatly improve spatial domain partition. In addition, the performance of m2ST2 is inferior to the m2ST3, suggesting that the masked mechanism aids the model learn more discriminative representations. Finally, m2ST is optimal in all cases, which indicates that all three proposed mechanisms contribute to improved spatial domain partition.

**Table 1.** The ablation study results on ARI metric (The bold values indicate the best results).

Methods	DLPFC (mean±SD)	Breast cancer	STARmap
m2ST1	0.1017 ± 0.0808	0.4400	0.2436
m2ST2	0.1392 ± 0.1176	0.4602	0.1000
m2ST3	0.4163 ± 0.0817	0.5757	0.5347
m2ST4	0.1635 ± 0.0584	0.4334	0.1974
m2ST	**0.5654 ± 0.0720**	**0.6475**	**0.6100**

### 3.4 The impact of different masked rates and scale numbers

To investigate the effect of different rates of masking and different scales on m2ST, we conducted experiments on the Breast Cancer, STARmap, and DLPFC-151672 datasets. The experimental results are shown in Supplementary Figs S13 and S14. As seen in Supplementary Fig. S13, the trends across the three metrics are consistent, with the best performance observed when the rate of masking is between 0.5 and 0.7. Moreover, Supplementary Fig. S13a demonstrates that when the masking rate is either too high or too low, the performance of the method deteriorates. This suggests that the appropriate rate of the masked feature vectors can enhance the robustness of the model. Then, as shown in Supplementary Fig. S14, the spatial domain partitioning demonstrates suboptimal performance when the number of scales is set to 1. With an increasing number of scales, the partitioning performance improves. However, it is also evident that more scales do not necessarily lead to better results. For example, the performance with 5 scales shows no significant improvement compared to 2 scales. Consequently, selecting 2–3 scales emerges as a robust choice for modeling.

## 4 Conclusion

In this study, we propose m2ST, a novel dual multi-scale graph clustering framework consisting of a multi-scale graph autoencoder and a multi-scale clustering method. Extensive experiments on five spatial transcriptomic datasets demonstrate m2ST’s superior performance over existing methods. Despite its strengths, m2ST still has several limitations. It relies on a pre-constructed adjacency matrix, which can be memory-intensive for large datasets, and separating adjacency matrix construction from spatial domain partitioning may lead to sub-optimal results. Additionally, treating multi-scale embedding learning and clustering as separate steps limits accuracy, and the model’s dependence on hyperparameters, such as learning rate and masking rate, affects robustness. These limitations will be addressed in our further work.

## Supplementary Material

btaf221_Supplementary_Data
