# Supplementary material for: m2ST: dual multi-scale graph clustering for spatially resolved transcriptomics
Source: Bioinformatics. 2025 Apr 24;41(5):btaf221. doi: 10.1093/bioinformatics/btaf221 (PMC12085222; doi:10.1093/bioinformatics/btaf221)
Supplement: btaf221_Supplementary_Data [file btaf221_supplementary_data.docx]

**Supplementary Materials for the manuscript “m2ST: Dual multi-scale graph clustering for spatially resolved transcriptomics”**

**Part 1 The graph construction method**

The adjacency matrix and feature matrix are crucial for graph construction. For the initial adjacency matrix $\mathbf{A}_{0}$, we first follow (Fang, et al., 2024) to calculate the Euclidean distance between sample points. To balance spatial information and the gene expression of individual cells, we also adopt the method in (Li, et al., 2022) and introduce a hyperparameter $\lambda>0$ to generate an enhanced hybrid adjacency matrix $\mathbf{A}$.

$\mathbf{A}=\left( 1-\lambda\right)\mathbf{A}_{0}+\lambda\mathbf{I}$ (S1)

where $\boldsymbol{I\in}R^{N\times N}$ is the identity matrix and *N* is the number of total instances of dataset.

For the feature matrix $\mathbf{X}$, we follow (Li, et al., 2022), and initialize it with the raw gene count data. The process primarily includes filtering out lowly expressed and low-variance genes, normalizing the counts for each cell, and performing batch correction when necessary.

| 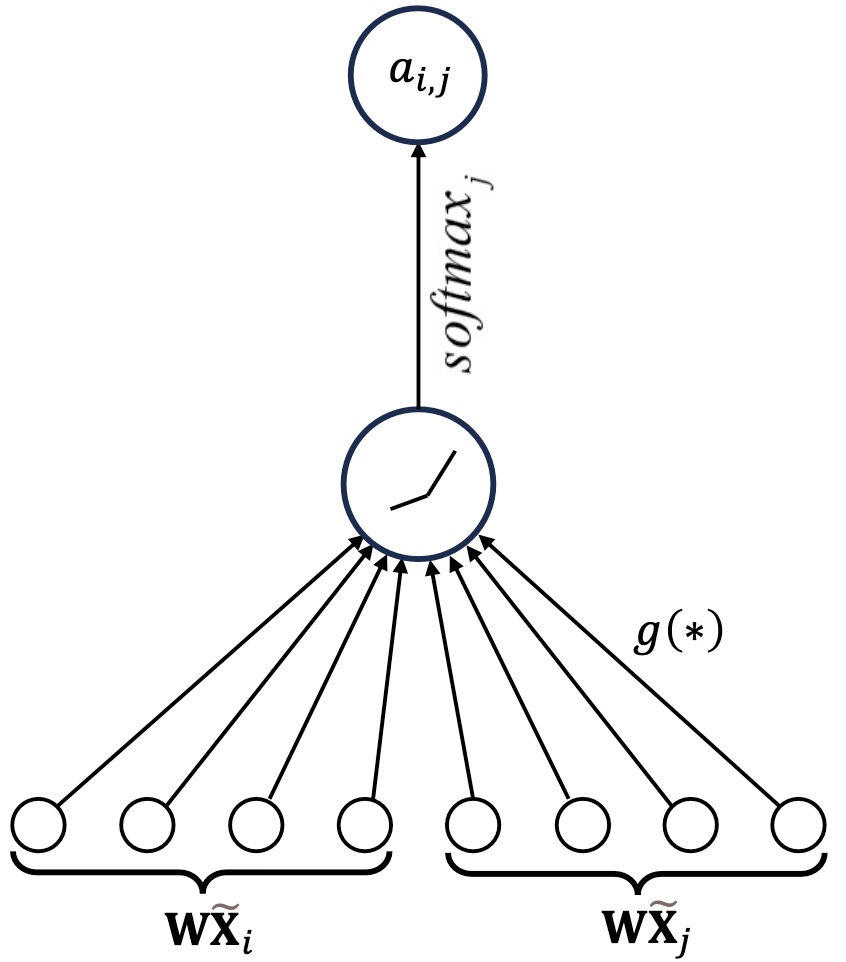 | 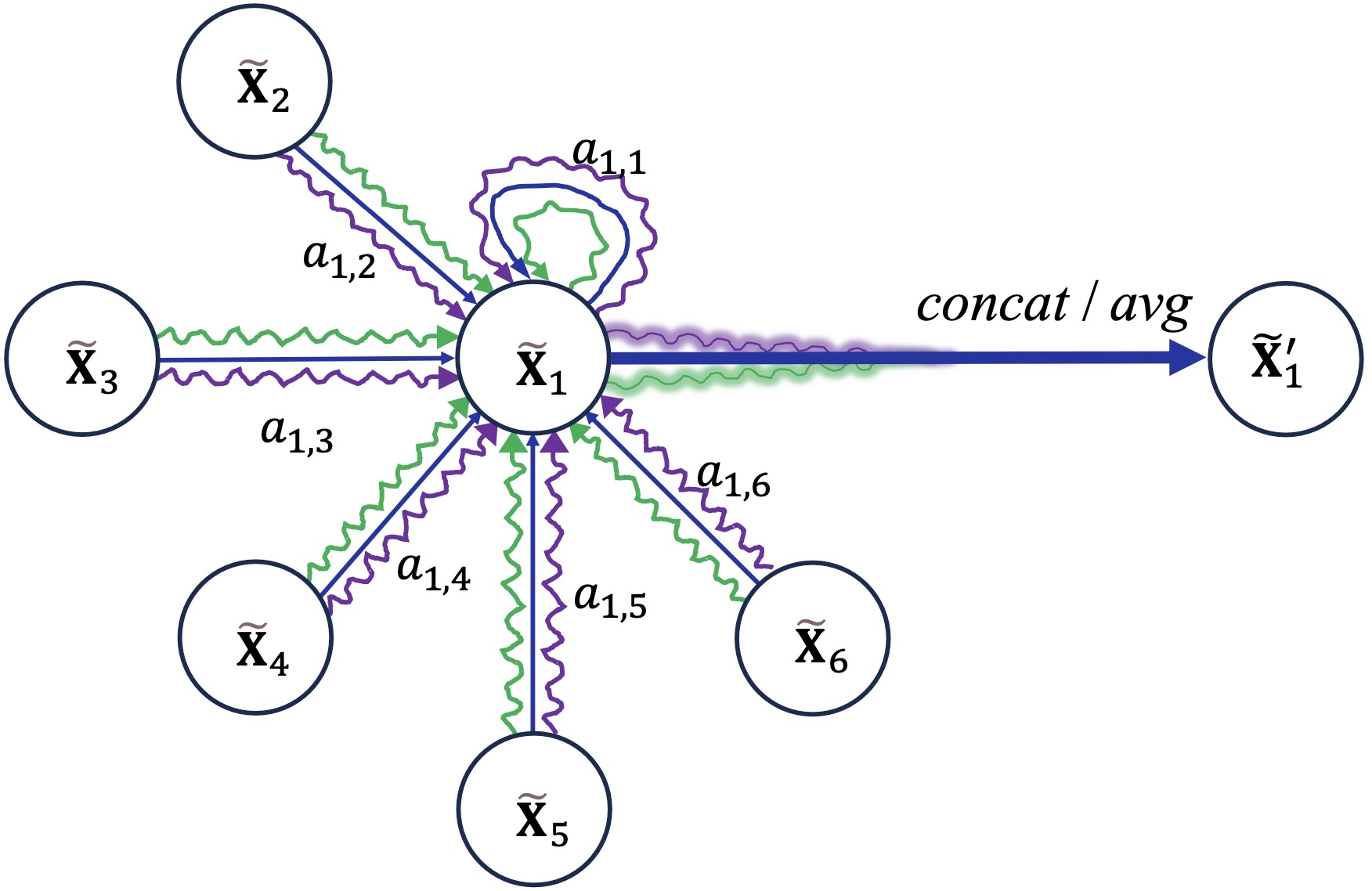 |
| --- | --- |
| (a) | (b) |

**Fig. S1**. Diagram of GAT. (a) Illustration of attention computation. (b) Illustration of node feature aggregation.

**Part 2 The detailed description and optimization process for multi-scale clustering**

Further details about (12) are explained below:

1. The first term $\sum_{m}^{M} \left\| \mathbf{H}^{m}-\mathbf{H}_{\boldsymbol{c}}^{T}\mathbf{W}^{m}-\mathbf{H}_{\boldsymbol{s}}^{m,T}\mathbf{P}^{m} \right\|_{F}^{2}$ in (12) is developed from traditional matrix factorization and is used to learn the common representation between views and specific representation of each view.
2. The second and third terms$\left\| \mathbf{H}^{T} \right\|_{F}^{2}$, $\sum_{k=1}^{K} \left\| \mathbf{S}^{k,T} \right\|_{F}^{2}$ in (12) are used to ensure robustness of the learned common and specific representations against noise and outliers.
3. The fourth and fifth terms $\sum_{m}^{M} \alpha^{m}\left\| \mathbf{H}_{\boldsymbol{s}}^{m}-\mathbf{V}^{m}\mathbf{U} \right\|_{F}^{2}$, $\alpha^{M+1}\left\| \mathbf{H}_{c}-\mathbf{V}^{M+1}\mathbf{U} \right\|_{F}^{2}$ in (12) are used to cluster the common representation $\mathbf{H}_{c}$ and the specific representation $\mathbf{H}_{\boldsymbol{s}}^{m}$.
4. The sixth term $\sum_{m}^{M+1} \left\| \mathbf{V}^{m,T}\mathbf{V}^{m}-\mathbf{I} \right\|_{F}^{2}$ in (12) is the orthogonal constraint for clustering center matrix. Previous works proved that the diversity within each view can be captured by using the orthogonality constraint on it (Liang, et al., 2020; Wang, et al., 2017). Therefore, we also introduced it to enhance the clustering center matrix of each view and further improve the clustering performance.
5. The seventh term $\sum_{m}^{M+1} \alpha^{m}\ln\alpha^{m}$ in (12) is the entropy of the weights of different views, which is used to adaptively adjust the importance of the views adaptively.

Then, an alternating optimization scheme is used to solve the problem in (12). First, by computing the partial derivatives of $\mathbf{H}$, $\mathbf{S}^{m}$, $\mathbf{W}^{m}$, $\mathbf{P}^{m}$, $\mathbf{V}^{m}$, and $\alpha^{m}$ and setting them to zero, the corresponding update formulas are derived as follows:

$\mathbf{H}=\left( \left( \gamma+\alpha^{M+1} \right)\mathbf{I}+\mathbf{W}^{m}\mathbf{W}^{m,T} \right)^{-1}\left( \sum_{m=1}^{M} \left( \mathbf{W}^{m}\mathbf{X}^{m,T}-\mathbf{W}^{m}\mathbf{P}^{m,T}\mathbf{S}^{m} \right)+\alpha^{M+1}\mathbf{V}^{M+1}\mathbf{U} \right)$ (S2)

$\mathbf{S}^{m}=\left( \left( \gamma+\alpha^{m} \right)\mathbf{I}+\mathbf{P}^{m}\mathbf{P}^{m,T} \right)^{-1}\left( \mathbf{P}^{m}\mathbf{X}^{m,T}-\mathbf{P}^{m}\mathbf{W}^{m,T}\mathbf{H}+\alpha^{m}\mathbf{V}^{m}\mathbf{U} \right), m=1,2,\ldots M$ (S3)

$\mathbf{W}^{m}=\left( \mathbf{H}\mathbf{H}^{T} \right)^{-1}\left( \mathbf{H}\mathbf{X}^{m}-\mathbf{H}\mathbf{S}^{m,T}\mathbf{P}^{m} \right), m=1,2,\ldots,M$ (S4)

$\mathbf{P}^{m}=\left( \mathbf{S}^{m}\mathbf{S}^{m,T} \right)^{-1}\left( \mathbf{S}^{m}\mathbf{X}^{m}-\mathbf{S}^{m}\mathbf{H}^{T}\mathbf{W}^{m} \right), m=1,2,\ldots,M$ (S5)

$a^{m}=\frac{exp\left( -{\left\| \mathbf{S}^{m}-\mathbf{V}^{m}\mathbf{U} \right\|_{F}^{2}}/\delta\right)}{\sum_{l=1}^{M+1} exp\left( -{\left\| \mathbf{S}^{l}-\mathbf{V}^{l}\mathbf{U} \right\|_{F}^{2}}/\delta\right)}, m=1,2,\ldots,M+1$ (S6)

$\mathbf{V}^{m}=\left( \alpha^{m}\mathbf{S}^{m}\mathbf{U}^{T} \right)\left( \alpha^{m}\mathbf{U}\mathbf{U}^{T}+\beta\mathbf{V}^{m,T}\mathbf{V}^{m}-\beta\mathbf{I} \right)^{-1}, m=1,2,\ldots,M+1$ (S7)

where $\mathbf{I}$ is the identity matrix.

For the partition matrix $\mathbf{U}$, we update it by calculating the distance between instances and cluster centers within each representation and combining these with the weights of each representation, as detailed below:

$\mathbf{U}_{i,j}\boldsymbol{=}\left\{ \begin{aligned} 1, i=\underset{c}{\mathrm{argmin}} \sum_{k=1}^{M+1} \alpha^{m}\left\| \mathbf{S}_{:,j}^{m}-\mathbf{V}_{:,c}^{m} \right\|_{F}^{2} \\ 0, otherwise \end{aligned} \right.$ (S8)

By iteratively implementing (S2)-(S8), the local optimal solution can be obtained.

**Part 3 Algorithm Description**

| **Algorithm 1 m2ST (Training)** |
| --- |
| **Input**: Spatial transcription graph data $G=\left( \mathcal{V,}\mathbf{A},\mathbf{X} \right)$; the number of epochs, the dimension of hidden representation $d_{m}^{'}$, $d^{'}$; the number of multi-head attentions *K*; scaling factor $\gamma$.  **While** epoch < Maxepoch  $\tilde{\mathbf{X}}\boldsymbol{\leftarrow}\mathrm{mask}\mathbf{X}with token \left[ \mathrm{Masked} \right]$  # Encoder part  ${\tilde{\mathbf{X}}}_{se}\boldsymbol{\leftarrow}f_{E}\left( \mathbf{A,}\tilde{\mathbf{X}} \right)$ $⊳$ Obtaining the primary embedding.  **For** *m*=1, 2, …, *M*  $\mathbf{H}^{m}\boldsymbol{\leftarrow}f_{E\_m}\left( \mathbf{A,}{\tilde{\mathbf{X}}}_{se} \right)$ $⊳$ Obtaining the multi-scale latent embeddings.  **End for**  # Decoder part  **For** *m*=1, 2, …, *M*  ${\tilde{\mathbf{H}}}^{m}\boldsymbol{\leftarrow}\mathrm{remask}\mathbf{H}^{m} with token \left[ \mathrm{Masked} \right]$  $\mathbf{Z}^{m}\boldsymbol{\leftarrow}f_{D\_m}\left( \mathbf{A}\boldsymbol{,}{\tilde{\mathbf{H}}}^{m} \right)$ $⊳$ Reconstructing latent embedding with different scales.  **End for**  # Computing loss  $loss\boldsymbol{\leftarrow}\mathcal{L}_{SCE}\left( \mathbf{X}_{i}\boldsymbol{,}\mathbf{Z}_{i}^{m} \right), i\in\tilde{\mathcal{V}}, m=1, 2, \ldots, M$;  Using Adam algorithm to update parameters.  **End while**  **Return** encoder and decoder model, loss. |

| **Algorithm 1 m2ST (Inference)** |
| --- |
| **Input**: Spatial transcription graph data $G=\left( \mathcal{V,}\mathbf{A},\mathbf{X} \right)$; the dimension of specified and common representations $d_{s}$ and $d_{c}$, maximum number of iterations *T*, balance parameters$\beta$, $\lambda$ and $\delta$.  # Encoder part  $\mathbf{X}_{se}\boldsymbol{\leftarrow}f_{E}\left( \mathbf{A,X} \right)$ $⊳$ Obtaining the primary embedding by using the trained shared encoder.  **For** *m*=1, 2, …, *M*  $\mathbf{H}^{m}\boldsymbol{\leftarrow}f_{E\_m}\left( \mathbf{A,}\mathbf{X}_{se} \right)$ $⊳$ Obtaining the multi-scale latent embeddings by using the trained multi-scale encoder.  **End for**  # One-step multi-scale clustering part  **Initialization:** initialize $\left\{ \mathbf{H}_{s}^{m} \right\}$, $\left\{ \mathbf{W}^{m} \right\}$, $\left\{ \mathbf{P}^{m} \right\}$, $\left\{ \mathbf{V}^{m} \right\}$, $\mathbf{U}$ randomly and initialize $\alpha^{m}=1/(K+1)$.  **While** $t<T$ and $\left\vert Obj\left( t \right)-Obj\left( t-1 \right) \right\vert<1e-6$ $⊳$ $Obj\left( t \right)$ is calculated according to (11).  By iteratively implementing (S2)-(S8). $⊳$ Update $\mathbf{H}$, $\mathbf{S}^{m}$, $\mathbf{W}^{m}$, $\mathbf{P}^{m}$, $\mathbf{V}^{m}$, $\alpha^{m}$ and $\mathbf{U}$ sequentially.  **End while**  **Return** Spatial domain annotation matrix $\mathbf{U}$. |

This subsection presents the detailed algorithm description and the proposed m2ST is given in algorithm 1. First, during the self-supervised training phase of the masked multi-scale graph autoencoder, we construct graph data based on spatial location information and randomly select a certain proportion of nodes, replacing their node features with masked tokens. Then, the graph data with partially observed features is fed into the encoder to generate latent node embedding. In the decoding phase, we apply a re-masking operation to the selected nodes. Then, the decoder is applied to the re-masked graph to produce reconstructed features. Finally, Computing the error between the reconstructed node features and the original node features, and the Adam optimizer is employed to update the network parameters.

Once the masked graph autoencoder is trained, we no longer perform any masking operations and use the trained encoder to extract multi-scale embeddings for the spatial transcription data. The extracted multi-scale embeddings are subsequently input into the one-step multi-scale clustering method to achieve the spatial domain annotations.

**Part 4 Experiments setting**

**Table S1.** The statistics of datasets

| Datasets | Slices | Spots | Genes | Classes | Platform |
| --- | --- | --- | --- | --- | --- |
| DLPFC | 151507 | 4226 | 20494 | 7 | 10X Visium |
|  | 151508 | 4384 | 20083 | 7 |  |
|  | 151509 | 4789 | 20732 | 7 |  |
|  | 151510 | 4634 | 20475 | 7 |  |
|  | 151669 | 3661 | 20583 | 5 |  |
|  | 151670 | 3498 | 20338 | 5 |  |
|  | 151671 | 4110 | 21037 | 5 |  |
|  | 151672 | 4015 | 10725 | 5 |  |
|  | 151673 | 3639 | 21267 | 7 |  |
|  | 151674 | 3673 | 21897 | 7 |  |
|  | 151675 | 3592 | 20783 | 7 |  |
|  | 151676 | 3460 | 20806 | 7 |  |
| Breast cancer | Section_1 | 3798 | 36601 | 20 | 10X Visium |
| STARmap | X | 1207 | 1020 | 7 | STARmap |
| Mouse hippocampus | Puck_200115_08 | 41786 | 4000 | 10 | Slide-seqV2 |
| Mouse cerebellum | Puck_180430_6 | 25551 | 18671 | 8 | Slide-seq |
| Human Heart | Heart_nondiseased_section | 26366 | 377 | 18 | Xenium |

**1) Datasets**

1) DLPFC (Pardo, et al., 2022): This dataset is a human dorsolateral prefrontal cortex region consisting of 12 slices with spatial transcription data extracted using 10X Visium technology (Rao, et al., 2020). The data is manually labeled with five to seven domains, each with clear boundaries.

2) Breast cancer (Xu, et al., 2024): this dataset is spatial transcription data on human breast cancer sections collected using 10X Visium technology. The dataset is artificially annotated with 20 domains based on morphological images and gene expression profiles, including four morphological types, i.e., ductal carcinoma in situ/globular carcinoma in situ (DCIS/LCIS), invasive ductal carcinoma (IDC), tumor margin regions and healthy regions.

3) STARmap (Wang, et al., 2018): This data is spatial transcription data extracted using a combination of barcoding and imaging techniques on slices of mouse visual cortex. This dataset is artificially labeled at seven domains.

4) Mouse hippocampus (Palla, et al., 2022): This dataset is spatial transcription data on mouse hippocampal slices collected using Slide-seqV2 technology, which contains ten cell domains.

5) Mouse cerebellum (Rodriques, et al., 2019; Shang and Zhou, 2022): This dataset is spatial transcription data on mouse cerebellum slices collected using Slide-seq technology, which contains eight cell domains.

6) Human Heart ((Xue, et al., 2025): This dataset is spatial transcription data on human heart non-diseased slices collected using Xenium technology, which contains eighteen domains.

**2) Parameters setting**

In this subsection, we present the detailed model parameter settings. First, for the multi-scale autoencoder, the maximum number of epochs is set to 200, the number of heads in the multi-head attention mechanism is set to 4, the number of hidden units is set to 256, the learning rate is set to 0.005, the masking rate is set to 0.5, the number of scales is set to 2, and the output dimensions for each scale are set to 256 and 128, respectively. Second, for the multi-scale clustering, the number of iterations is set to 100, the dimensions of the common and specific representations are set within [10, 20, …, 100], the parameters$\beta$, $\lambda$ and $\delta$ are set within [1e-5, 1e-4, …, 1e5]. The optimal parameters and the dimensions of representations are determined by grid search following the existing clustering method (Ji and Feng, 2025; Xu, et al., 2019). For the DLPFC dataset the random seed is set to 0, for other datasets the random seed is set to [0,10,100,1000,10000]. The visualization results show the annotation results when the random seed is 0.

**3) The introduction to the evaluation indices**

Five performance indices, i.e., NMI, Purity, ARI, SC and DB, are used in the experiments to evaluate the algorithms. They are presented in detail as follows:

**1) Normalized Mutual Information (NMI)**: NMI measures the similarity between two clusters of the same instances and normalizes the similarity values. The calculation equation is as follows:

$\mathrm{NMI}=\frac{\sum_{i=1}^{c} \sum_{j=1}^{c} n_{ij}\log\left( {n\cdot n_{ij}}/{n_{i}\cdot n_{j}} \right)}{\sqrt{\sum_{i=1}^{c} n_{i}\log\left( {n_{i}}/n \right)\cdot\sum_{j=1}^{c} n_{j}\log\left( {n_{j}}/n \right)}}$ (S9)

where $n_{ij}$ is the number of instances belonging to the *i*th cluster and the *j*th class, $n_{i}$ and $n_{j}$ are the number of instances belonging to the *i*th cluster and *j*th cluster, respectively. *n* is the total number of instances.

**2) Purity**: Purity is a simple and transparent evaluation measure, which are given as follows:

$Purity=\sum_{i=1}^{c} \frac{n_{i}}{n}P\left( S_{i} \right)$ (S10)

where *c* is the number of clusters, *n* is the total number of the instances, and $n_{i}$ is the number of instances of the *i*th cluster. $P\left( S_{i} \right)$ is defined as follows:

$P\left( S_{i} \right)=\frac{1}{n_{i}}\max_{j} \left( n_{i}^{j} \right)$ (S11)

where $n_{i}^{j}$ is the number of the *i*th input class that is assigned to the *j*th cluster.

**3) Adjusted Rand Index (ARI)**: ARI is the corrected-for-chance version of the rand index (RI), which is given as follows:

$ARI=\frac{RI-E\left( RI \right)}{max\left( RI \right)-E\left( RI \right)}$ (S12)

$RI=\frac{f_{00}+f_{11}}{{n(n-1)}/2}$ (S13)

where *E*(*RI*) is the expectation of RI, $f_{00}$ is the number of instance pairs with different real class labels and belonging to different clusters, $f_{11}$ is the number of instance pairs with the same class labels and belonging to the same cluster, and *n* is the number of instances in the whole dataset.

**4) Davies–Bouldin index (DB)**: DBI is a function of the ratio of the within-cluster scatter to the between-cluster separation, which is defined as follows:

$\mathrm{DBI}=\frac{1}{c}\sum_{l=1}^{c} \max_{j,j\neq l} \left\{ \frac{s_{l}+s_{j}}{\left\| \mathbf{V}_{l}-\mathbf{V}_{j} \right\|^{2}} \right\}$ (S14)

$s_{l}=\frac{1}{n_{l}}\sum_{\mathbf{x}_{i}\in c_{l}} \left\| \mathbf{x}_{i}-\mathbf{V}_{l} \right\|^{2}$ (S15)

where $n_{l}$ is the number of instances in cluster *l*, and $c_{l}$ is the set of instances in the *l-*th cluster.

**5) Silhouette Coefficient index (SC)**: SC is a function to measure the difference between the similarity of each instance to an instance in the same cluster and the similarity to an instance in the nearest neighboring cluster.

$SC=\frac{1}{N}\sum_{i=1}^{N} \frac{b_{i}-a_{i}}{max(b_{i},a_{i})}$ (S16)

where $a_{i}$ is the average distance from the *i*-th sample to all samples in the same cluster, and $b_{i}$ is the average distance from *i*-th sample to all samples in the cluster closest to it.

**Part 5 Experimental results**


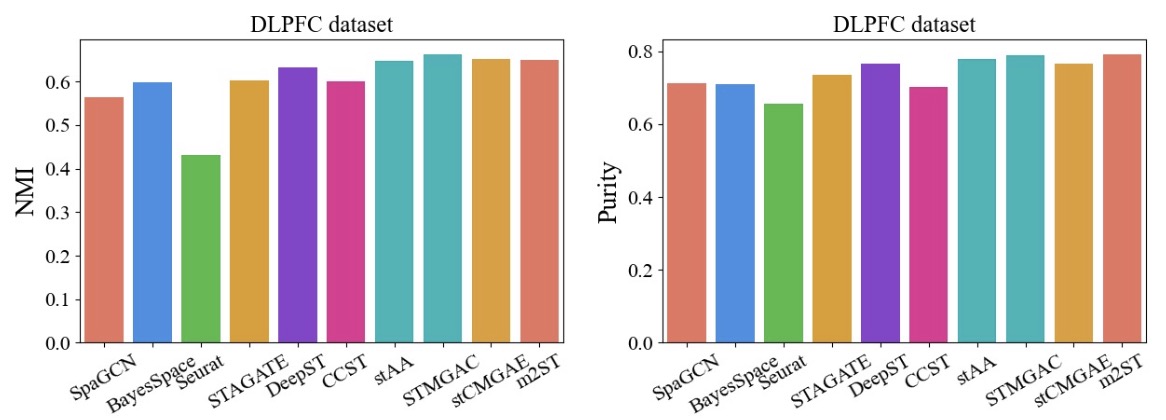


**Fig. S2**. Clustering results for all methods on DLPFC on NMI and Purity metrics.


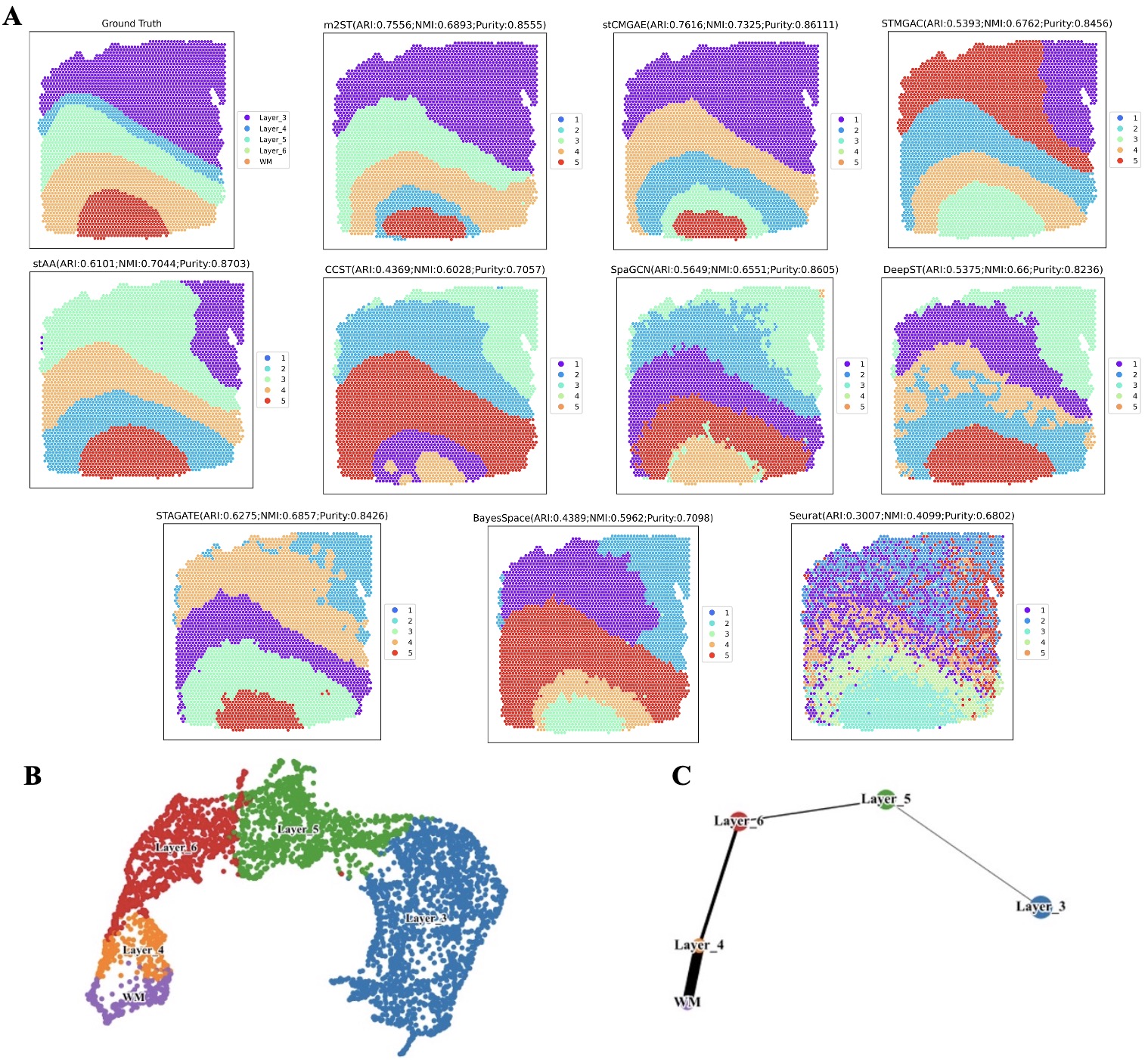


**Fig. S3**. Comparison of the proposed m2ST and other methods on the DLPFC dataset. (A) Experimental results of visualization of ground truth and all methods on slice 151672. (B) and (C) Visualization results of UMAP plot and spatial trajectory inference of the proposed m2ST on the slice 151672, respectively.


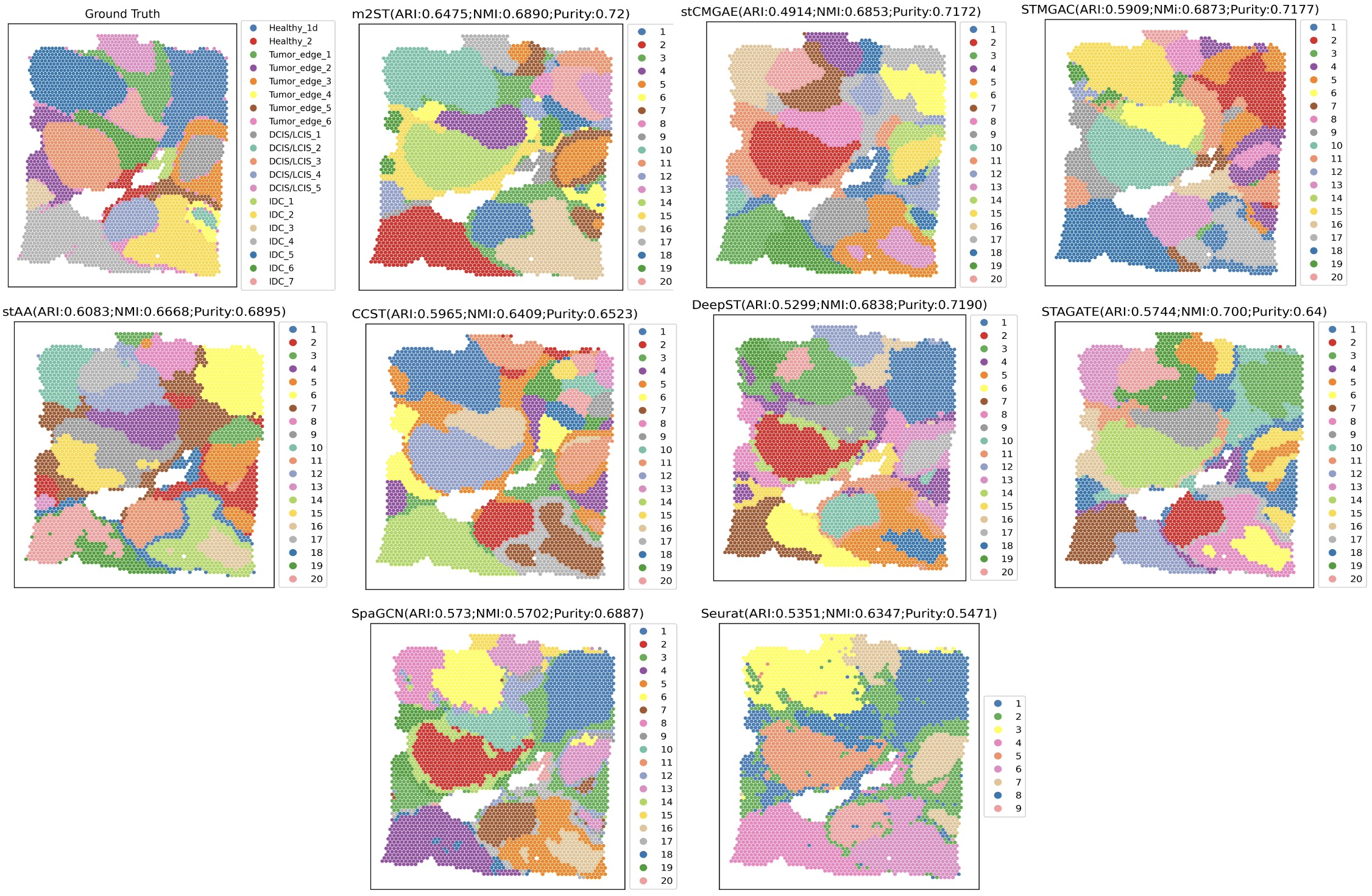


**Fig. S4.** Visualization results of ground true and all methods on Breast cancer dataset and Seruat can only cluster 9 domains.


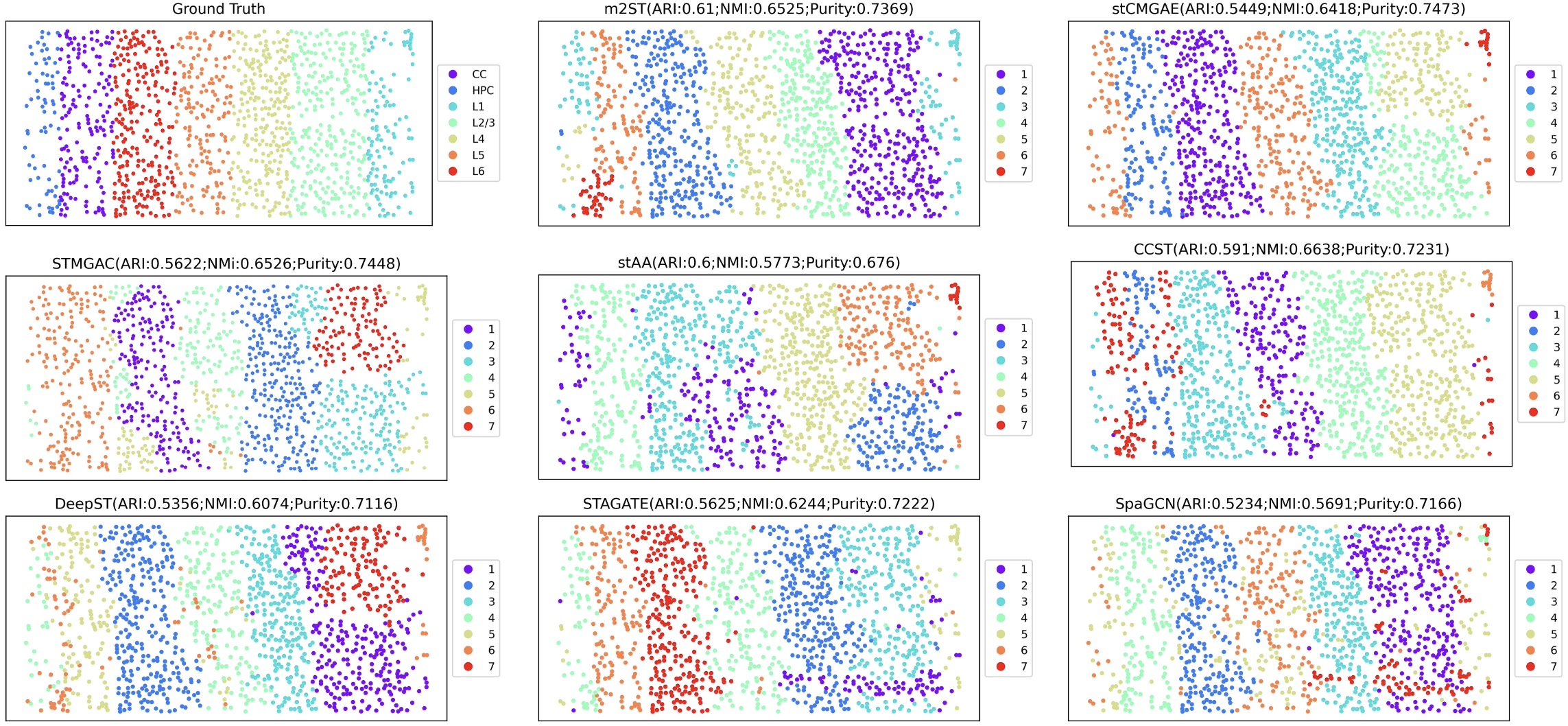


**Fig. S5.** Visualization results of ground true and all methods on STARmap dataset.


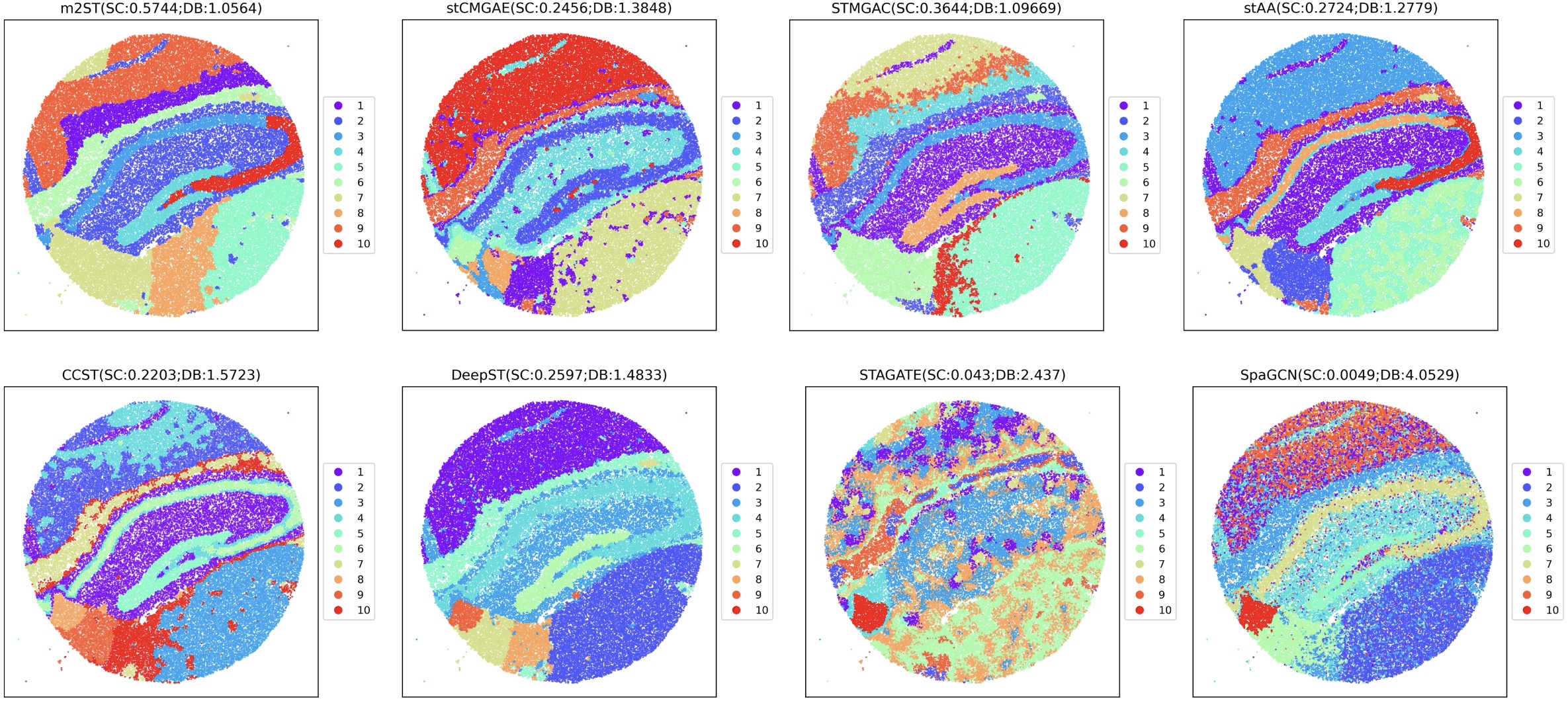


**Fig. S6.** Visualization results of ground true and all methods on Mouse hippocampus dataset.


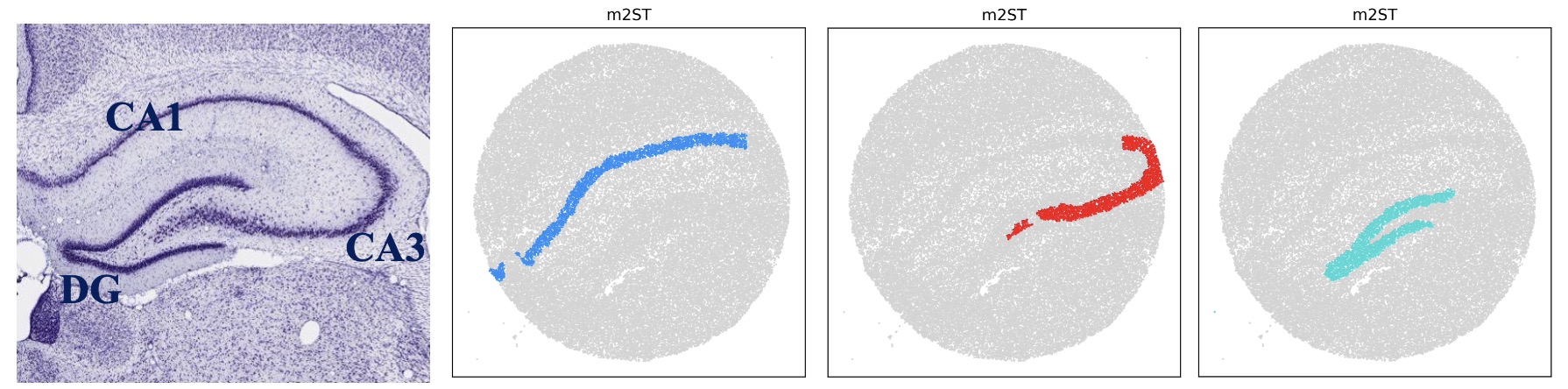


**Fig. S7.** The laminar structure of the mouse hippocampus is provided by the Allen Reference Atlas (Coronal Atlas), and three domains clustered by m2ST are shown on each spatial location (CA1, CA3, and DG).


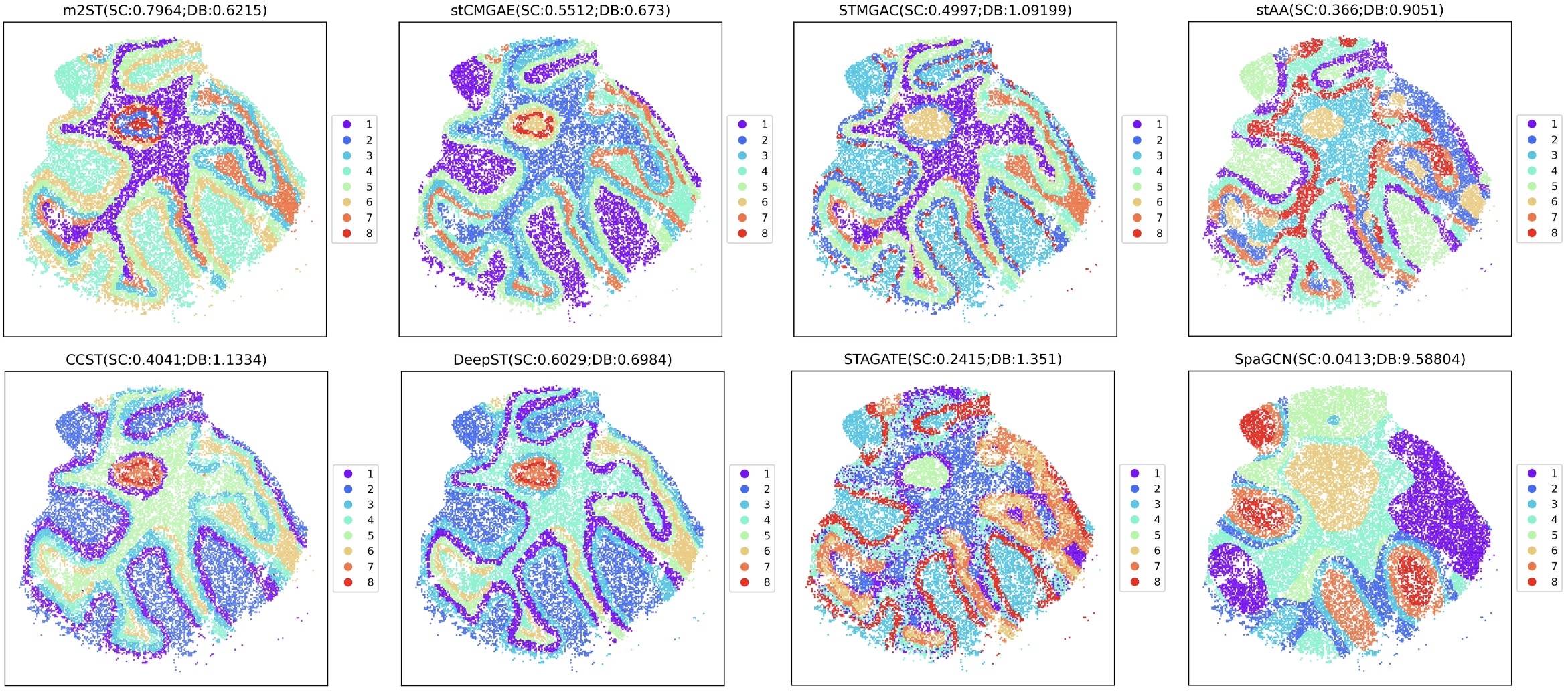


**Fig. S8.** Visualization results of ground true and all methods on Mouse cerebellum dataset.


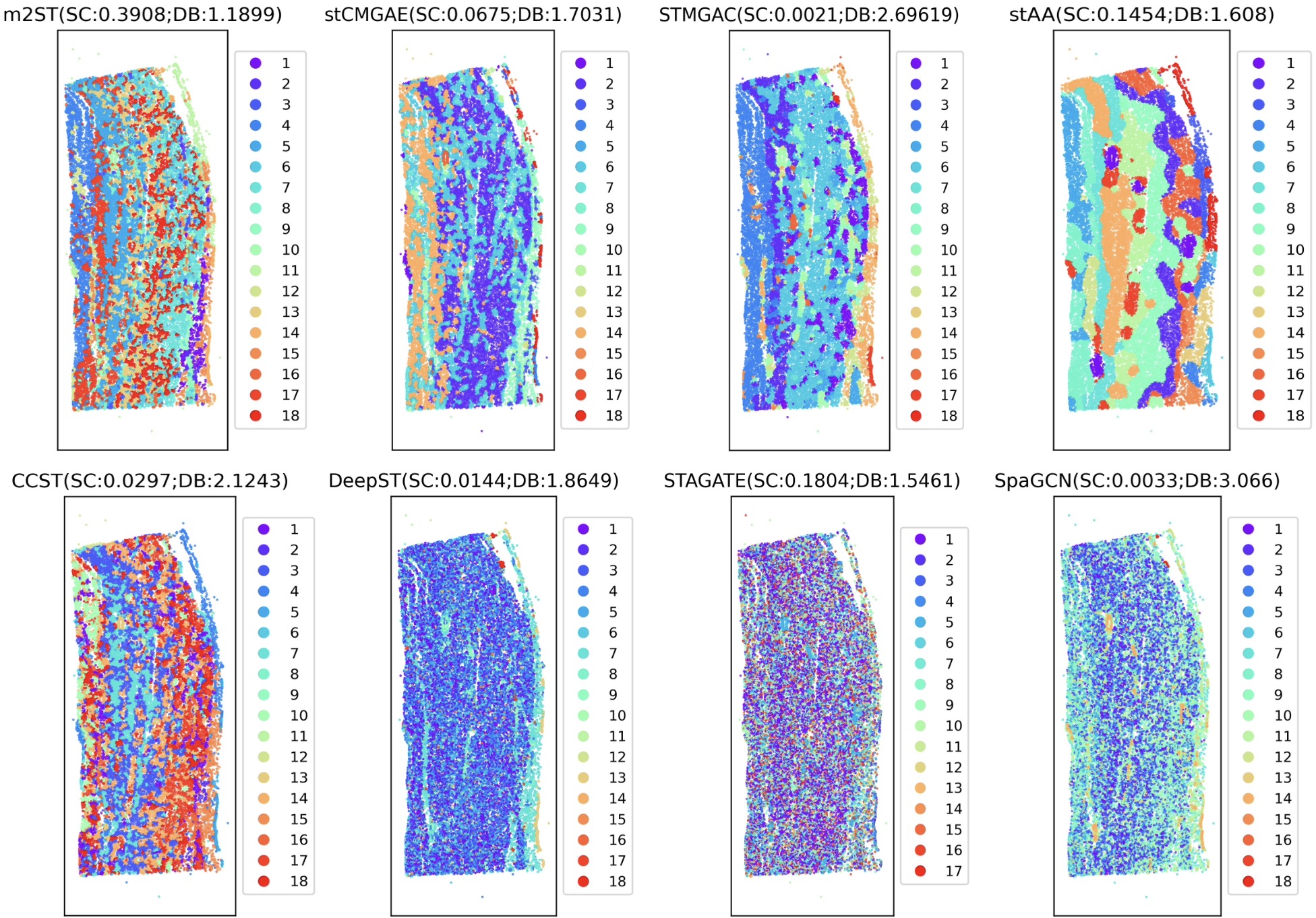


**Fig. S9.** Visualization results of ground true and all methods on Human Heart dataset.


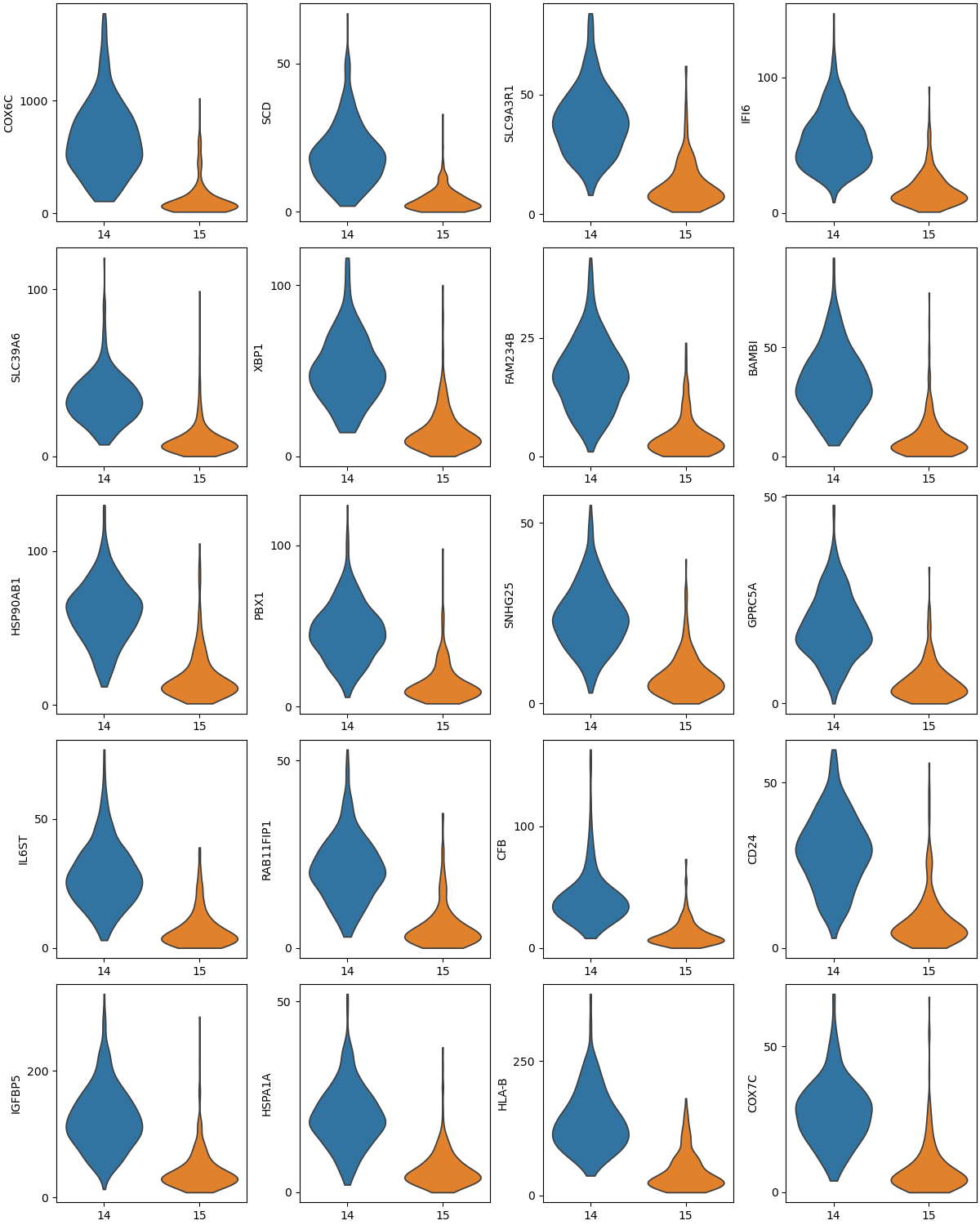


**Fig. S10.** The differential gene expression analysis between the 14 and 15 clusters of the Breast cancer dataset.


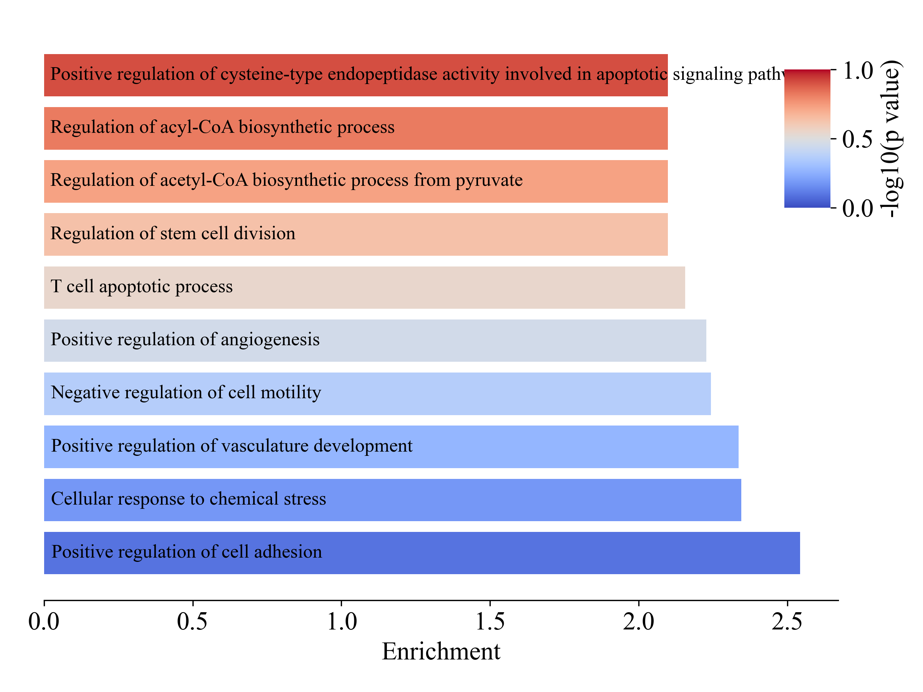


**Fig. S11.** The enriched GO terms in cluster 15 of the Breast cancer dataset.


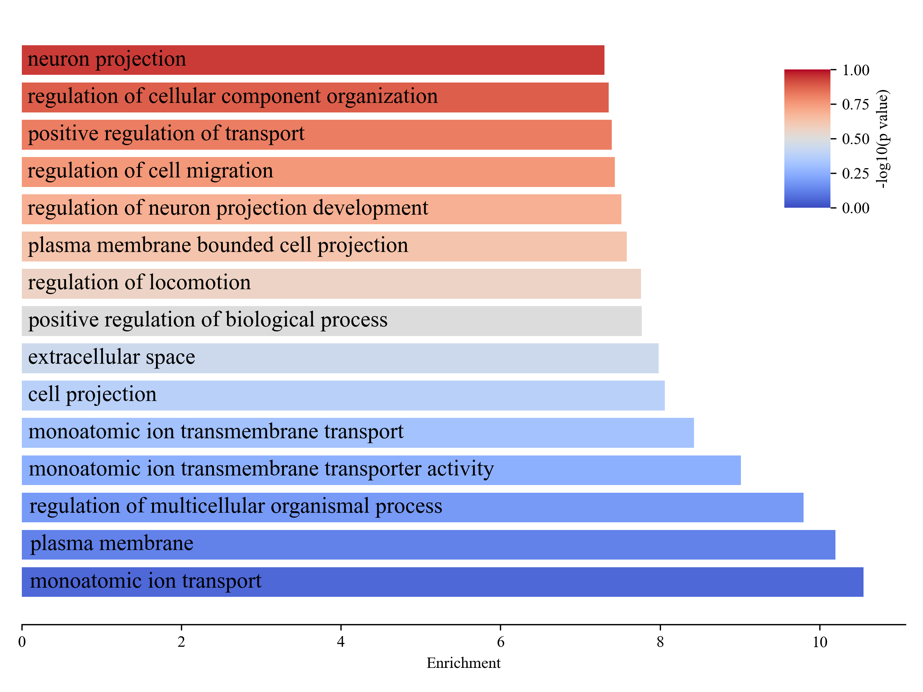


**Fig. S12.** The enriched GO terms in cluster 8 of the Mouse cerebellum dataset.

**Table S2.** The ablation study results on NMI and Purity metrics

| Methods | DLPFC (mean±std) | | Breast cancer | | STARmap | |
| --- | --- | --- | --- | --- | --- | --- |
|  | NMI | Purity | NMI | Purity | NMI | Purity |
| m2ST1 | 0.2175±0.0544 | 0.4833±0.0932 | 0.5831 | 0.6203 | 0.3310 | 0.4598 |
| m2ST2 | 0.3086±0.0911 | 0.5382±0.0654 | 0.5203 | 0.5573 | 0.2315 | 0.3893 |
| m2ST3 | 0.6084±0.0601 | 0.7152±0.0817 | 0.6593 | 0.6782 | 0.6333 | 0.7481 |
| m2ST4 | 0.1995±0.0481 | 0.5160±0.0846 | 0.5378 | 0.5949 | 0.2963 | 0.4614 |
| m2ST | **0.6405±0.0930** | **0.7865±0.0356** | **0.6890** | **0.7162** | **0.6525** | **0.7639** |

* The bolded indicates the optimal result

| 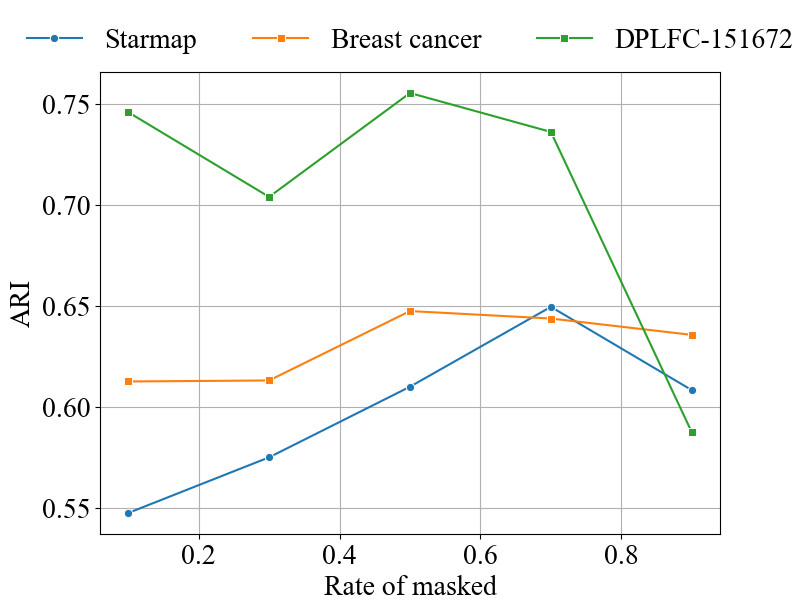 | 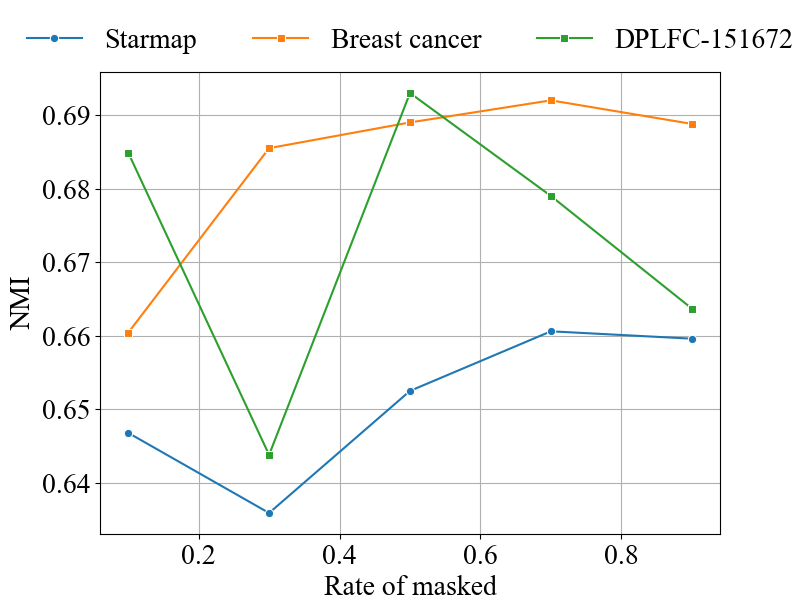 | 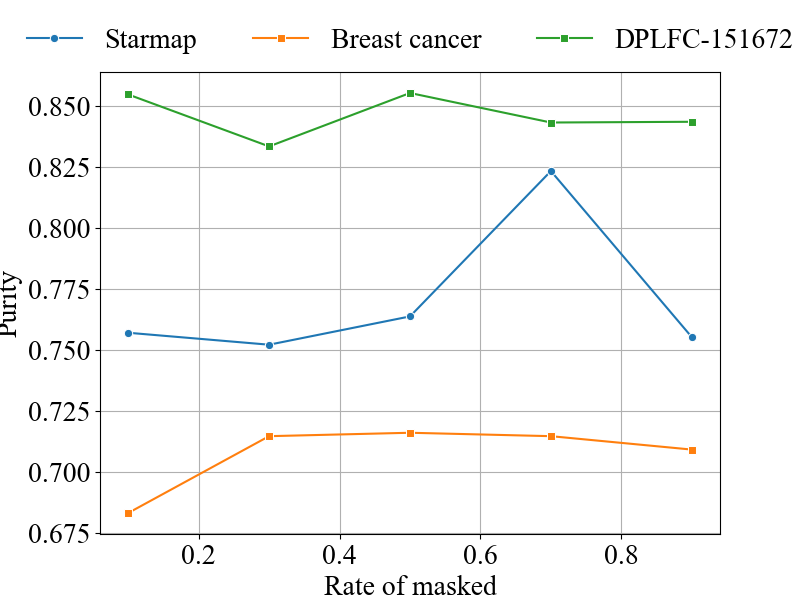 |
| --- | --- | --- |
| (a) | (b) | (c) |

**Fig. S13.** Impacts of different rate of masked

| 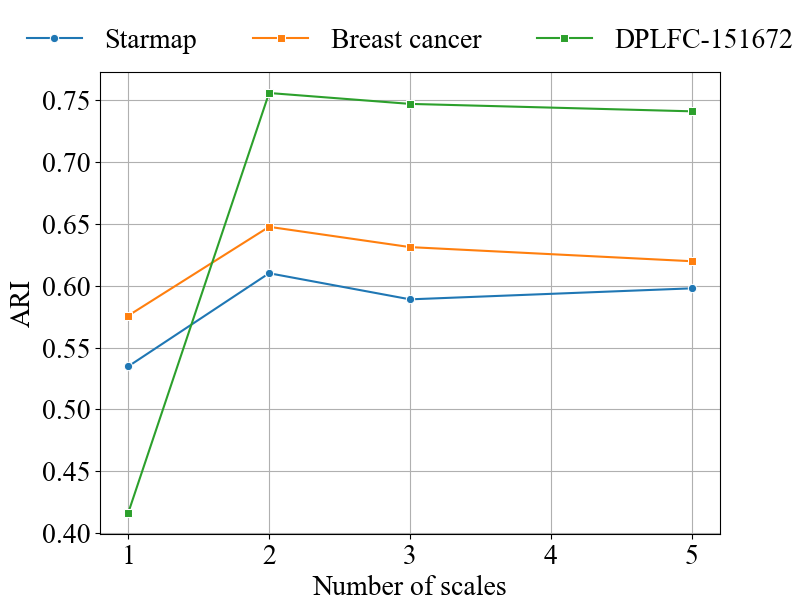 | 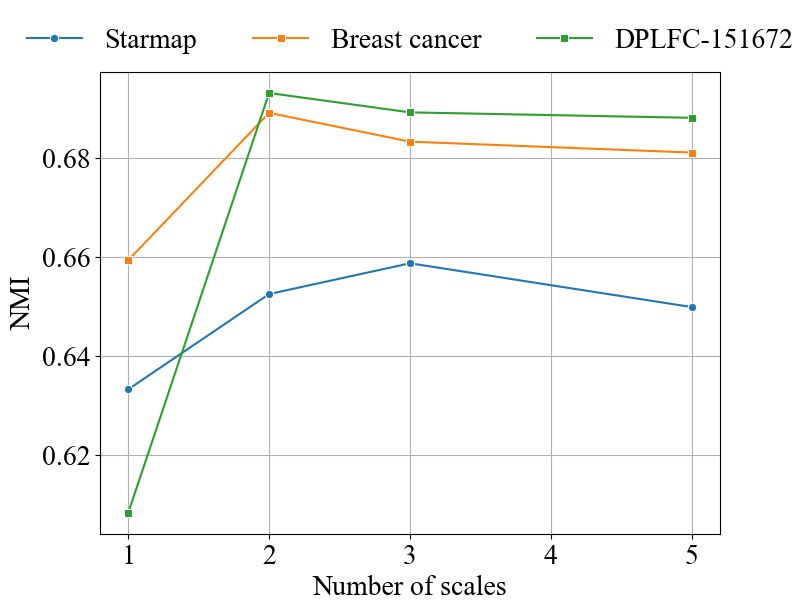 | 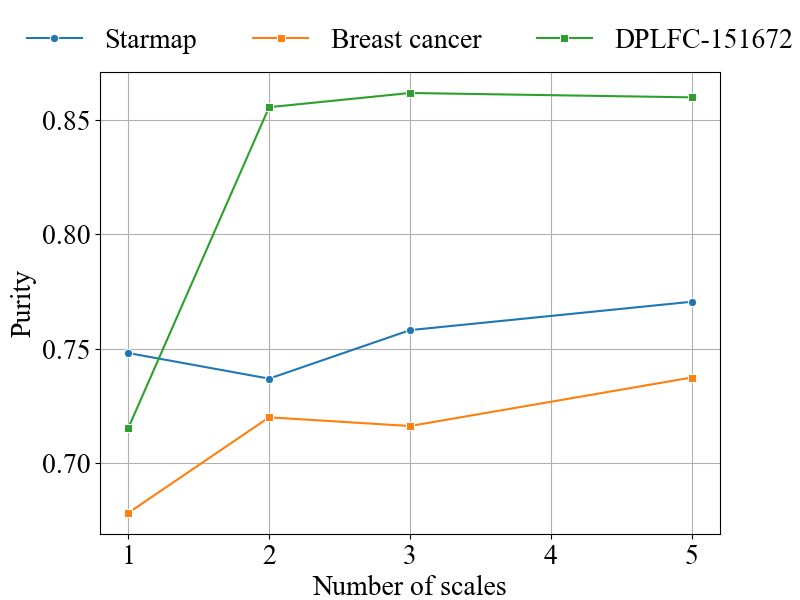 |
| --- | --- | --- |
| (a) | (b) | (c) |

**Fig. S14.** Impacts of different number of scales

References

Fang, Z., Liu, T., Zheng, R., *et al.* (2024) stAA: adversarial graph autoencoder for spatial clustering task of spatially resolved transcriptomics. *Briefings in Bioinformatics,* **25**, bbad500.

Ji, J. and Feng, S. (2025) Anchors Crash Tensor: Efficient and Scalable Tensorial Multi-view Subspace Clustering. *IEEE Transactions on Pattern Analysis and Machine Intelligence* DOI: 10.1109/TPAMI.2025.3526790.

Li, J.*, et al.* (2022) Cell clustering for spatial transcriptomics data with graph neural networks. *Nature Computational Science*, **2**, 399-408.

Liang, N.*, et al.* (2020) Multi-view clustering by non-negative matrix factorization with co-orthogonal constraints. *Knowledge-Based Systems*, **194**, 105582.

Palla, G.*, et al.* (2022) Squidpy: a scalable framework for spatial omics analysis. *Nature methods*, **19**, 171-178.

Pardo, B.*, et al.* (2022) spatialLIBD: an R/Bioconductor package to visualize spatially-resolved transcriptomics data. *BMC genomics*, **23**, 434.

Rao, N., Clark, S. and Habern, O., (2020) Bridging genomics and tissue pathology: 10x genomics explores new frontiers with the visium spatial gene expression solution. *Genetic Engineering & Biotechnology News*, **40**, 50-51.

Rodriques, S.G.*, et al.* (2019) Slide-seq: A scalable technology for measuring genome-wide expression at high spatial resolution. *Science*, **363**, 1463-1467.

Shang, L. and Zhou, X. (2022) Spatially aware dimension reduction for spatial transcriptomics. *Nature communications*, **13**, 7203.

Wang, J.*, et al.* (2017) Diverse non-negative matrix factorization for multiview data representation. *IEEE Transactions on Cybernetics*, **48**, 2620-2632.

Wang, X.*, et al.* (2018) Three-dimensional intact-tissue sequencing of single-cell transcriptional states. *Science*, **361**, eaat5691.

Xu, H.*, et al.* (2024) Unsupervised spatially embedded deep representation of spatial transcriptomics. *Genome Medicine*, **16**, 12.

Xu, P.*, et al.* (2019) Multi-view information-theoretic co-clustering for co-occurrence data. In, *Proceedings of the AAAI conference on artificial intelligence* 2019, 379-386.

Xue, S.*, et al.* (2025) Inferring single-cell resolution spatial gene expression via fusing spot-based spatial transcriptomics, location, and histology using GCN. *Briefings in Bioinformatics*, **26**, bbae630.
